# Maximum Matchings in Geometric Intersection Graphs

**DOI:** 10.1007/s00454-023-00564-3

**Published:** 2023-09-09

**Authors:** Édouard Bonnet, Sergio Cabello, Wolfgang Mulzer

**Affiliations:** 1 Université de Lyon, CNRS, ENS de Lyon, Université Claude Bernard Lyon 1, LIP UMR5668, Lyon, France; 2 Department of Mathematics, Faculty of Mathematics and Physics, University of Ljubljana, Ljubljana, Slovenia; 3 Department of Mathematics, Institute of Mathematics, Physics and Mechanics, Ljubljana, Slovenia; 4 Institut für Informatik, Freie Universität Berlin, Berlin, Germany

**Keywords:** Computational geometry, Geometric intersection graph, Disk graph, Unit-disk graph, Matching, 68Q25, 68W40

## Abstract

Let *G* be an intersection graph of *n* geometric objects in the plane. We show that a maximum matching in *G* can be found in $O\hspace{0.33325pt}(\rho ^{3\omega /2}n^{\omega /2})$ time with high probability, where $\rho$ is the density of the geometric objects and $\omega >2$ is a constant such that $n\times n$ matrices can be multiplied in $O(n^\omega )$ time. The same result holds for any subgraph of *G*, as long as a geometric representation is at hand. For this, we combine algebraic methods, namely computing the rank of a matrix via Gaussian elimination, with the fact that geometric intersection graphs have small separators. We also show that in many interesting cases, the maximum matching problem in a general geometric intersection graph can be reduced to the case of bounded density. In particular, a maximum matching in the intersection graph of any family of translates of a convex object in the plane can be found in $O(n^{\omega /2})$ time with high probability, and a maximum matching in the intersection graph of a family of planar disks with radii in $[1, \Psi ]$ can be found in $O\hspace{0.33325pt}(\Psi ^6\log ^{11}\hspace{-0.55542pt}n + \Psi ^{12 \omega } n^{\omega /2})$ time with high probability.

## Introduction

Let $\mathscr {U}$ be a family of (connected and compact) objects in ${\mathbb {R}}^2$. The *intersection graph* $G_\mathscr {U}$ of $\mathscr {U}$ is the undirected graph with vertex set $\mathscr {U}$ and edge set

$$\begin{aligned}E(G_\mathscr {U}) = \{ UV \mid U,V \in \mathscr {U}, \, U \cap V \ne \emptyset \}.\end{aligned}$$

If the objects in $\mathscr {U}$ are partitioned into two sets, one can also define the *bipartite* intersection graph, a subgraph of $G_\mathscr {U}$, in the obvious way. Consider the particular case when $\mathscr {U}$ is a set of disks. Then, we call $G_\mathscr {U}$ a *disk graph*, and if all disks in $\mathscr {U}$ have the same radius, a *unit-disk graph*. Unit disk graphs are often used to model ad-hoc wireless communication networks and sensor networks [13, 16, 36]. Disks of varying sizes and other shapes become relevant when different sensors cover different areas. Moreover, general disk graphs serve as a tool to approach other problems, like the barrier resilience problem [20].

We consider a classic optimization problem, *maximum matching*, in the setting of geometric intersection graphs, and introduce two new techniques, each interesting in its own. First, we provide an efficient algorithm to compute a maximum matching in any subgraph of the intersection graph of geometric objects of low density. Second, we provide a sparsification technique to reduce the maximum matching problem in a geometric intersection graph to the case of low density. The sparsification works for convex shapes of similar sizes for which certain range searching operations can be done efficiently.

We use $\omega$ to denote a constant such that $\omega > 2$ and any two $n \times n$ matrices can be multiplied in time $O(n^\omega )$.^1^

*Maximum matching in intersection graphs of geometric objects of low density*   We first introduce some geometric concepts. The diameter of a set $X \subset {\mathbb {R}}^2$, denoted by ${{\,\mathrm{diam\hspace{0.44434pt}}\,}}(X)$, is the supremum of the distances between any two points of *X*. The *density* $\rho (\mathscr {U})$ of a family $\mathscr {U}$ of objects is

1 $$\begin{aligned} \rho (\mathscr {U})=\max _{X \subseteq {\mathbb {R}}^2}\, |\{U \in \mathscr {U}\mid {{\,\mathrm{diam\hspace{0.44434pt}}\,}}(U) \geqslant {{\,\mathrm{diam\hspace{0.44434pt}}\,}}(X), \, U \cap X \ne \emptyset \}|. \end{aligned}$$

One can also define the density by considering for *X* only disks. Since an object of diameter *d* can be covered by *O*(1) disks of diameter *d*, this changes the resulting parameter by only a constant. (See, for example, the book by de Berg et al. [6, Sect. 12.5] for such a definition.) The *depth* (ply) of $\mathscr {U}$ is the largest number of objects that cover a single point:

$$\begin{aligned}\max _{p \in {\mathbb {R}}^2}\, |\{ U\in \mathscr {U}\mid p\in U\}|.\end{aligned}$$

For disk graphs and square graphs, the depth and the density are linearly related; see for example Har-Peled and Quanrud [15, Lem. 2.7]. More generally, bounded depth and bounded density are equivalent whenever we consider homothets of a constant number of shapes. Density and depth are usually considered in the context of realistic input models; see de Berg et al. [7] for a general discussion.

Let $\mathbb {G}_\rho$ be the family of *subgraphs* of intersection graphs of geometric objects in the plane with density at most $\rho$.^2^ Our goal is to compute a maximum matching in graphs of $\mathbb {G}_\rho$, assuming the availability of a geometric representation of the graph and a few basic geometric primitives on the geometric objects. For this, we consider the density $\rho$ as an additional parameter. Naturally, the case $\rho = O(1)$ of *bounded density* is of particular interest.

In a general graph $G=(V,E)$ with *n* vertices and *m* edges, the best running time for computing a maximum matching in *G* depends on the ratio *m*/*n*. The classic algorithm of Micali and Vazirani [25, 33] is based on augmenting paths, and it finds a maximum matching in $O\hspace{0.33325pt}(m\sqrt{n})$ time. Mucha and Sankowski [27] use algebraic tools to achieve running time $O(n^\omega )$. As we shall see, for $G\in \mathbb {G}_\rho$, we have $m = O(\rho n)$, and this bound is asymptotically tight. Thus, for $G \in \mathbb {G}_\rho$, the running times of these two algorithms become $O\hspace{0.33325pt}(\rho n^{3/2})$ and $O(n^\omega )$, respectively.

In general *bipartite* graphs, a recent algorithm by Mądry [24] achieves running time roughly $O(m^{10/7})$.^3^ Efrat et al. [11] show how to compute the maximum matching in bipartite unit disk graphs in $O\hspace{0.30548pt}(n^{3/2}\log n)$ time. Having bounded density does not help in this algorithm; it has $O(\sqrt{n})$ rounds, each of which needs $\Omega (n)$ time. The same approach can be used for other geometric shapes if a certain semi-dynamic data structure is available. In particular, using the data structure of Kaplan et al. [19] for additively-weighted nearest neighbors, finding a maximum matching in a bipartite intersection graph of disks takes $O\hspace{0.30548pt}(n^{3/2}{{\,\textrm{polylog}\,}}n)$ time. We are not aware of any similar results for non-bipartite geometric intersection graphs.

We show that a maximum matching in a graph of $\mathbb {G}_\rho$ with *n* vertices can be computed in $O\hspace{0.31097pt}(\rho ^{3\omega /2} n^{\omega /2})=O\hspace{0.30548pt}(\rho ^{3.56} n^{1.19})$ time. The algorithm is randomized and succeeds with high probability. It uses the algebraic approach by Mucha and Sankowski [28] for planar graphs with an extension by Yuster and Zwick [35] for *H*-minor-free graphs. As noted by Alon and Yuster [4], this approach works for *hereditary*^4^ graph families with bounded average degree and small separators. We note that the algorithm can be used for graphs of $\mathbb {G}_\rho$, because we have average degree $O(\rho )$ and balanced separators of size $O(\sqrt{\rho n})$ [15, 31]. However, finding the actual dependency on $\rho$ is difficult because it plays a role in the average degree, in the size of the separators, and because the algorithm has a complex structure with several subroutines that must be distilled.

There are several noteworthy features in our approach. For one, we solve a geometric problem using linear algebra, namely Gaussian elimination. The use of geometry is limited to finding separators, bounding the degree, and constructing the graph explicitly. Note that the role of subgraphs in the definition of $\mathbb {G}_\rho$ is a key feature in our algorithm. On the one hand, we need a hereditary family of graphs, as needed to apply the algorithm. On the other hand, it brings more generality; for example, it includes the case of bipartite graphs defined by colored geometric objects.

Compared to the work of Efrat et al. [11], our algorithm is for arbitrary subgraphs of geometric intersection graphs, not only bipartite ones; it works for any objects, as it does not use advanced data structures that may depend on the shapes. On the other hand, it needs the assumption of low density. Compared to previous algorithms for arbitrary graphs and ignoring polylogarithmic factors, our algorithm is faster when $\rho = o\hspace{0.33325pt}(n^{(20-7\omega )/(21\omega -20)})$. Using the current bound $\omega < 2.373$, this means that our new algorithm is faster for $\rho = O(n^{0.113})$.

Our matching algorithm also applies for intersection graphs of objects in 3-dimensional space. However, in this case there is no algorithmic gain with the current bounds on $\omega$: one gets a running time of $O(n^{2\omega /3})$ when $\rho =O(1)$, which is worse than constructing the graph explicitly and using the algorithm of Micali and Vazirani.

*Sparsification—Reducing to bounded depth*   Consider a family of convex geometric objects $\mathscr {U}$ in the plane where each object contains a square of side length 1 and is contained in a square of side length $\Psi \geqslant 1$. Our objective is to compute a maximum matching in the intersection graph $G_\mathscr {U}$.^5^ Our goal is to transform this problem to finding a maximum matching in the intersection graph of a subfamily $\mathscr {U}'\subset \mathscr {U}$ with bounded depth. Then we can employ our result from above for $G_{\mathscr {U}'}$ or, more generally, any algorithm for maximum matching (taking advantage of the sparsity of the new instance).

We describe a method that is fairly general and works under comparatively mild assumptions and also in higher dimensions. However, for an efficient implementation, we require that the objects under consideration support certain range searching operations efficiently. We discuss how this can be done for disks of arbitrary sizes, translates of a fixed convex shape in the plane, axis-parallel objects in constant dimension, and (unit) balls in constant dimension. In all these cases, we obtain a subquadratic time algorithm for finding a maximum matching, assuming that $\Psi$ is small. We mostly focus on the planar case, mentioning higher dimensions as appropriate.

As particular results to highlight, we show that a maximum matching in the intersection graph of any family of translates of a convex object in the plane can be found in $O(n^{\omega /2})$ time with high probability, and a maximum matching in the intersection graph of a family of planar disks with radii in $[1, \Psi ]$ can be found in $O\hspace{0.33325pt}(\Psi ^6\log ^{11}\hspace{-0.55542pt}n + \Psi ^{12 \omega } n^{\omega /2})$ time with high probability. See Table 1 for a summary of the results in this context.

**Table 1:** Time complexity to compute the maximum matching in an intersection graph

Objects	Time complexity	Reference
Disks of radius in $[1,\Psi ]$ in ${\mathbb {R}}^2$	$O\hspace{0.33325pt}(\Psi ^6 n \log ^{11}\hspace{-0.7222pt}n + \Psi ^{12\omega } n^{\omega /2})$	Theorem 5.3
Translates of *O*(1) convex objects in ${\mathbb {R}}^2$	$O(n^{\omega /2})$	Theorem 5.9
Axis-parallel rectangles in ${\mathbb {R}}^2$ with edges in $[1,\Psi ]$	$(1+\Psi )^{O(1)}n^{\omega /2}$	Theorem 5.11
Axis-parallel boxes in ${\mathbb {R}}^d$ with edges in $[1,\Psi ]$	$(1+\Psi )^{O(d)}n^{3/2}$	Corollary 5.12
Unit balls in ${\mathbb {R}}^3$ or ${\mathbb {R}}^4$	$O(n^{3/2})$	Theorem 5.14
Unit balls in ${\mathbb {R}}^d$, $d\geqslant 5$	$O\hspace{0.27771pt}\bigl (n^{2 \lceil d/2\rceil /(1+ \lceil d/2\rceil )+\varepsilon }\bigr )$	Theorem 5.14

In some cases, the result is correct with high probability

*Organization*   We begin with some general definitions and basic properties of geometric intersection graphs (Sect. 2). Then, in the first part of the paper, we present the new algorithm for finding a maximum matching in geometric intersection graphs of low density (Sect. 3). In the second part, we present our sparsification method. This is done in two steps. First, we describe a generic algorithm that works for general families of shapes that have roughly the same size, assuming that certain geometric operations can be performed quickly (Sect. 4). Second, we explain how to implement these operations for several specific shape families, e.g., translates of a given convex objects and disks of bounded radius ratio (Sect. 5). The two parts are basically independent, where the second part uses the result from the first part as a black box, to state the desired running times.

## Basics of (Geometric Intersection) Graphs

*Geometric objects and computational model*   Several of our algorithms work under fairly weak assumptions on the geometric input: we assume that the objects in ${\mathscr {U}}$ have *constant description complexity*. This means that the boundary of each object is a continuous closed curve whose graph is a semialgebraic set, defined by a constant number of polynomial equalities and inequalities of constant maximum degree. For later algorithms we restrict attention to some particular geometric objects, like disks or squares.

To operate on $\mathscr {U}$, we require that our computational model supports primitive operations that involve a constant number of objects of $\mathscr {U}$ in constant time, e.g., finding the intersection points of two boundary curves; finding the intersection points between a boundary curve and a disk or a vertical line; testing whether a point lies inside, outside, or on the boundary of an object; decomposing a boundary curve into *x*-monotone pieces, etc. See, e.g., [19] for a further discussion and justification of these assumptions.

We emphasize that in addition to the primitives on the input objects, we do not require any special constant-time operations. In particular, even though our algorithms use algebraic techniques such as fast matrix multiplication or Gaussian elimination, we rely only on algebraic operations over ${\mathbb {Z}}_p$, where $p=\Theta (n^4)$ is a prime. Thus, we work only with numbers of $O(\log n)$-bits, and assuming a standard unit-cost model for such word-sizes, as in, e.g., the word-RAM model of computation, we simply need to bound the number of arithmetic operations in our algorithms.

*Geometric intersection graphs*   The following well-known lemma bounds $|G_\mathscr {U}|$ in terms of $\rho$, and the time to construct $G_\mathscr {U}$. We include a proof for completeness.

### Lemma 1.1

If $\mathscr {U}$ has *n* objects and density $\rho$, then $G_\mathscr {U}$ has at most $(\rho - 1)\hspace{0.55542pt}n$ edges (this holds in any dimension). If ${\mathscr {U}}$ consists of objects in the plane, then $G_\mathscr {U}$ can be constructed in $O\hspace{0.30548pt}(\rho n \log n)$ time.

### Proof

The bound on $|E(G_\mathscr {U})|$ uses a simple and well-known trick; see, e.g., [15, Lemma 2.6]: we orient each edge in $G_\mathscr {U}$ from the object of smaller diameter to the object of larger diameter. Then, the out-degree of each $X \in \mathscr {U}$ is at most $\rho - 1$, since by (1), there are at most $\rho$ objects $U \in \mathscr {U}$ with ${{\,\mathrm{diam\hspace{0.44434pt}}\,}}(U) \geqslant {{\,\mathrm{diam\hspace{0.44434pt}}\,}}(X)$ that intersect *X*, with *X* being one of them.

Next, we describe the construction of $G_\mathscr {U}$ in the planar case; see [18] for a similar algorithm in the context of disk graphs. Set $k = |E(G_\mathscr {U})|$. If $U, V \in \mathscr {U}$ form an edge in $E(G_\mathscr {U})$, then either (i) their boundaries intersect; or (ii) one is contained inside the other. To find the edges of type (i), we perform a plane sweep [5, 6].^6^ For this, we split the boundary of each object into a constant number of *x*-monotone pieces. We sweep a vertical line $\ell$ across the plane, and we maintain the intersection of $\ell$ with the pieces of the boundary curves. The events are the start and end points of the pieces of the boundary curves, as well as their pairwise intersections. There are $O\hspace{0.30548pt}(n + k)$ events. When we detect a boundary-boundary intersection, we add the corresponding edge to the output. An edge can be added *O*(1) times, so we sort the output to remove duplicates. Thus, it takes $O((n + k)\log n)$ time to find all edges of type (i).

To find the edges of type (ii), we perform a second plane sweep to compute the *trapezoidal decomposition* of the planar arrangement defined by the objects in $\mathscr {U}$ [6]. The trapezoidal decomposition is obtained by shooting upward and downward vertical rays from each *x*-extremal point on a boundary curve and from each intersection between two boundary curves. The rays end once they encounter a boundary curve, or they go into infinity. This results in a subdivision of the plane into $O\hspace{0.30548pt}(n + k)$ (possibly unbounded) *pseudo-trapezoids*. The subdivision can be computed in $O\hspace{0.33325pt}((n + k)\log n)$ time. We construct the *dual graph* of the trapezoidal decomposition, in which the vertices are the pseudo-trapezoids, and two pseudo-trapezoids are adjacent if and only if their boundaries intersect in more than one point. We perform a DFS in the resulting dual graph, keeping track of the objects in $\mathscr {U}$ that contain the current pseudo-trapezoid. Whenever we enter an object $U \in \mathscr {U}$ for the first time, we generate all edges between the objects that contain the current pseudo-trapezoid and *U*. This takes $O(n + k)$ time. We generate all edges of type (ii), and we possibly rediscover some edges of type (i). Thus, we sort the output once more to remove duplicates. The total running time is $O\hspace{0.33325pt}((n + k)\log n) = O\hspace{0.33325pt}(\rho n \log n)$.

We remark that using more sophisticated methods, such as randomized incremental construction [29], it may be possible to improve the running time to $O\hspace{0.33325pt}(\rho n + n\log n)$. However, this will not help us, because later parts of the algorithm will dominate the running time. $\square$

*Separators in geometric intersection graphs*   The classic *planar separator theorem* by Lipton and Tarjan [9, 22] shows that any planar graph can be decomposed in a balanced way by removing a small number of vertices. Even though geometric intersection graphs can be far from planar, similar results are also available for them. These results are usually parameterized by the *depth* of the arrangement or by the *area* of the separator and the components [3, 12, 26]. The following recent result provides a small separator for general intersection graphs of bounded density.

### Theorem 1.2

[15, Lemma 2.21] Let $\mathscr {U}$ be a set of *n* objects in ${\mathbb {R}}^2$ with density $\rho$. In *O*(*n*) expected time, we can find a circle $\mathbb {S}$ such that $\mathbb {S}$ intersects at most $c\sqrt{\rho n}$ objects of $\mathscr {U}$, the exterior of $\mathbb {S}$ contains at most $\alpha n$ elements of $\mathscr {U}$, and the interior of $\mathbb {S}$ contains at most $\alpha n$ elements of $\mathscr {U}$. Here $0<c$ and $0<\alpha <1$ are universal constants, independent of $\rho$ and *n*.

The proof of Theorem 2.2 goes roughly as follows: Pick a point in each object of $\mathscr {U}$, compute the smallest circle $\mathbb {S}'$ (or an approximation thereof) that contains, say, *n*/20 points, and then take a concentric scaled copy $\mathbb {S}$ of $\mathbb {S}'$, with scale factor uniformly at random in [1, 2]. With constant probability, the circle $\mathbb {S}'$ has the desired property. This can be checked easily in linear time by determining which objects of $\mathscr {U}$ are inside, outside, or intersected by $\mathbb {S}$. In expectation, a constant number of repetitions is needed to obtain the desired circle.

A family $\mathbb {G}$ of graphs is *hereditary* if for every $G \in \mathbb {G}$, it holds that all subgraphs *H* of *G* are also in $\mathbb {G}$. By definition, our family $\mathbb {G}_\rho$ of subgraphs of geometric intersection graphs with density $\rho$ is hereditary. A graph *G* is $\delta$-*sparse* if every subgraph *H* of *G* has at most $\delta \hspace{0.33325pt}|V(H)|$ edges. Lemma 2.1 implies that all graphs in $\mathbb {G}_\rho$ are $\rho$-sparse.

**Fig. 1:**
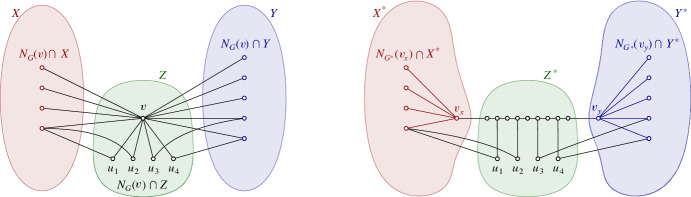
Splitting one single vertex of *Z*

Consider a graph *G* and a vertex *v* of *G*. A *vertex split* at *v* consists of adding a pendant 2-path $v v'v''$, where $v'$ and $v''$ are new vertices, and possibly replacing some edges *uv* incident to *v* by new edges $uv''$; see Fig. 1 for a sequence of splits. We note that a vertex split may not replace any edges. In this case, we are just adding a pendant path of length 2. Let $G'$ be a graph obtained from *G* by a sequence of *k* vertex splits. Then, the size of a maximum matching in $G'$ is the size of a maximum matching in *G* plus *k*. Furthermore, from a maximum matching in $G'$, we can easily obtain a maximum matching in *G* in $O\hspace{0.33325pt}(|V(G)| + |E(G)| + k)$ time. We will use vertex splits to ensure that the resulting graphs have bounded degree and a vertex set of a certain cardinality. Note that if we perform a vertex split at *v* in a graph of $\mathbb {G}_\rho$, in general we obtain a graph of $\mathbb {G}_{\rho +2}$ because we can represent it by making two new copies of the object corresponding to *v*. Nevertheless, this increase in the density will not be problematic in our algorithm.

## Maximum Matching in Low-Density Geometric Intersection Graphs

### Separators and Separator Trees

A graph *G* has a $(k, \alpha )$-separation if *V*(*G*) can be partitioned into three pairwise disjoint sets *X*, *Y*, *Z* such that $|X\cup Z|\leqslant \alpha \hspace{0.33325pt}|V(G)|$, $|Y\cup Z|\leqslant \alpha \hspace{0.33325pt}|V(G)|$, $|Z|\leqslant k$, and such that there is no edge with one endpoint in *X* and one endpoint in *Y*. We say that *Z* *separates* *X* and *Y*. At the cost of making the constant $\alpha$ larger, we can restrict our attention to graphs of a certain minimum size.

Theorem 2.2 gives a $(c\sqrt{\rho n},\alpha ')$-separation for every graph of $\mathbb {G}_\rho$, for some constant $\alpha ' < 1$. (A separator in $G_\mathscr {U}$ is a separator in every subgraph of $G_\mathscr {U}$ that is obtained by omitting edges from $G_\mathscr {U}$.) Furthermore, such a separation can be computed in expected linear time, if the objects defining the graph are available.

A recursive application of separations can be represented as a binary rooted tree. We will use so-called (weak) *separator trees*, where the separator does not go into the subproblems. In such a tree, we store the separator at the root and recurse on each side to obtain the subtrees. We want to have small separators and balanced partitions at each level of the recursion, and we finish the recursion when we get to problems of a certain size. This leads to the following definition. Let $\gamma > 0$, $0< \beta < 1$, and $0< \alpha <1$ be constants. We say that a graph *G* has a $(\gamma ,\beta ,\alpha )$-*separator tree* if there is a rooted binary full tree *T* with the following properties:

- Each node $t \in T$ is associated with some set $Z_t \subseteq V(G)$.
- The sets $Z_t$, $t\in T$, partition *V*(*G*), i.e., $\bigcup _{t\in T} Z_t =V(G)$, and $Z_t\cap Z_{t'}=\emptyset$, for distinct $t,t'\in T$.
- For each node $t \in T$, let $V_t = \bigcup _s Z_s$, where *s* ranges over the descendants of *t* (including *t*). Note that if *t* is an internal node with children *u* and *v*, then $V_t$ is the disjoint union of $Z_t$, $V_u$, and $V_v$. If *t* is a leaf, then $V_t = Z_t$.
- For each internal node $t \in T$ with children *u* and *v*, $(V_u, V_v, Z_t)$ is a $(\gamma m^\beta ,\alpha )$-separation for $G[V_t]$, the subgraph of *G* induced by $V_t$, where $m = |V_t| = |Z_t| + |V_u| + |V_v|$.
- For each leaf $t \in T$, we have $|V_t| = \Theta (\gamma ^{1/(1-\beta )})$. We have chosen the size so that $V_t$ is a $(\gamma \hspace{0.31097pt}|V_t|^\beta ,\alpha )$-separator for the whole induced subgraph $G[V_t]$.

Yuster and Zwick [35] provide an algorithm that computes a separator tree of some split graph for a given graph from an *H*-minor-free family. As Alon and Yuster [4, Lemma 2.13] point out, this algorithm actually works for any $\delta$-sparse hereditary graph family, as long as $\delta$ is constant. Thus, the result applies to $\mathbb {G}_\rho$. We revise the construction to make the dependency on $\rho$ explicit.

#### Lemma 1.3

Given a graph *G* of $\mathbb {G}_\rho$ with *n* vertices, we can compute in $O(\rho n\log n)$ expected time a vertex-split graph $G'$ of *G* and a separator tree $T'$ for $G'$ with the following properties:

- the graph $G'$ has $\Theta (\rho n)$ vertices and edges;
- the maximum degree of $G'$ is at most 4;
- $T'$ is a $(\gamma =O(\rho ),\beta =1/2, \alpha )$-separator tree for $G'$, where $\alpha <1$ is a constant (independent of $\rho$ and *n*).

#### Proof

We adapt the construction of Yuster and Zwick [35, Lemma 2.1], with three main changes: First, Yuster and Zwick assume $(\,{\cdot }, \alpha = 2/3)$-separations, but this specific value of $\alpha$ is not needed. Second, we make “dummy additions” of vertices to obtain a more balanced separation, which we later use as a black box. (Otherwise, we would get a $(\,{\cdot }, \,{\cdot }, O(1/\rho ))$-separator tree, and we would have to analyze the tree more carefully to obtain the same final result.) Third, we work out the constants in the analysis to understand the dependency on the density $\rho$.

We proceed recursively. Consider an *m*-vertex graph $G \in \mathbb {G}_\rho$ that appears during the recursion. If $m \leqslant C\rho$, where *C* is a sufficiently large constant, we make a sequence of vertex splits to reduce the maximum degree to three. This may increase the number of vertices. To ensure that this number is uniform, we add a (possibly empty) pendant path of even length until we get the maximum possible number of $\Theta (\rho ^2)$ vertices. The resulting separator tree $T'$ consists of a single node.

Now suppose that $m > C \rho$. Using Theorem 2.2, we get a $(c \sqrt{\rho m},\alpha )$-separation (*X*, *Y*, *Z*) of *G*. Thus, $|Z|\leqslant c \sqrt{\rho m}$. Yuster and Zwick [35, Lemma 2.1] explain how to make vertex splits at the vertices of *Z* and how to redefine the separation so that the vertices of the separator have maximum degree three. See Fig. 1 for how to split a vertex $v\in Z$. After making the vertex splits of Fig. 1 in all vertices of *Z*, we get a split graph $G^*$ of *G* and a separation $(X^*,Y^*,Z^*)$ with

- $$|X|\leqslant |X^*|\leqslant |X|+ |Z| \leqslant \alpha m$$,
- $$|Y|\leqslant |Y^*|\leqslant |Y|+ |Z| \leqslant \alpha m$$,
- $$|Z^*|\leqslant 4\hspace{0.44434pt}|Z|+6\hspace{0.44434pt}|E(G[Z])| \leqslant (4 + 6\rho ) |Z| = O(\rho ^{3/2} m^{1/2})$$ (by Lemma 2.1),
- vertices of $Z^*$ have degree at most 3 in $G^*$,
- $G^*[X^*]$ and $G^*[Y^*]$ are isomorphic to subgraphs of *G*, i.e., $G^*[X^*]$ is isomorphic to $G[X\cup Z]$ minus the edges of *G*[*Z*], and $G^*[Y^*]$ is isomorphic to $G[Y \cup Z]$ minus the edges of *G*[*Z*].

We recurse on $G[X^*]$ and $G[Y^*]$, each of which lies in $\mathbb {G}_\rho$, as it is (isomorphic to) a subgraph of *G*. In particular, the density of the graphs encountered during the recursion does not increase.

The recursive call on $G^*[X^*]$ yields a graph $G'_X$ and a tree $T'_X$, and the recursive call on $G^*[Y^*]$ yields a graph $G'_Y$ and a tree $T'_Y$, both with the properties given in the theorem. Let $G'$ be the graph obtained by putting together $G^*[Z^*]$, $G'_X$, and $G'_Y$. If some vertex degree gets larger than four, we can make a vertex split there. There are at most $|Z^*|$ such vertex splits. A separator tree $T'$ for $G'$ is constructed by making a root for $Z^*$ and making the roots of $T'_X$ and $T'_Y$ its two children. Adding a pendant path of even length to $G^*[Z^*]$, if needed, we ensure that $|Z^*| = \Theta (\rho |Z|)$, the maximum number of possible vertices after the splits (we again denote the resulting vertex set by $Z^*$).

For a graph *G* with *m* vertices considered during the recursion, by Theorem 2.2, we spend $\Theta (m)$ expected time to find the separation (*X*, *Y*, *Z*). Then, we construct the induced graph *G*[*Z*] in $O(\rho \hspace{0.27771pt}|Z|)=O(\rho ^{3/2} m^{1/2})$ time. The transformation from *G* to $G^*$ can be done in $O(\rho |Z|)=O(\rho ^{3/2} m^{1/2})$ time. Finally, when we may add vertices to $|Z^*|$, we spend $\Theta (\rho m)$ time. Standard tools to analyze recursions imply that the expected running time is distributed evenly over the $O(\log n)$ levels of the tree. Thus, the expected total running time is $O\hspace{0.33325pt}(\rho n \log n)$.

It is easy to see that the number of vertices of $G'$ is $\Theta (\rho m)$ because $G'_X$ has $\Theta (\rho |X^*|)$ vertices, $G'_Y$ has $\Theta (\rho |Y^*|)$ vertices, and $G[Z^*]$ has $\Theta (\rho |Z|)$ vertices. Since $G'$ has bounded maximum degree, it also has $\Theta (\rho m)$ edges. Furthermore, $(V(G'_X),V(G'_Y),Z^*)$ is a separation of $G'$. Since $G'$ has $\Theta (\rho m)$ vertices, $G'_X$ has $\Theta (\rho |X^*|)=\Theta (\rho \hspace{0.27771pt}|X|)$ vertices, and (*X*, *Y*, *Z*) is a separation of *G*, $|V(G'_X)| \leqslant \alpha ' m$ for some constant $\alpha '<1$. The same argument applies to $|V(G'_Y)|$. Since

$$\begin{aligned}|Z^*|=\Theta (\rho |Z|)= O(\rho \sqrt{\rho m}) =\Theta \bigl (\rho \sqrt{|V(G')|}\bigr ),\end{aligned}$$

it follows that $T'$ is a $(O(\rho ),\beta =1/2, \alpha ')$-separator tree. $\square$

Note that the split graph $G'$ in Lemma 3.1 is not necessarily in $\mathbb {G}_\rho$. It is a subgraph of an intersection graph, but since we introduce copies of geometric objects when we split vertices, the density increases. In any case, this does not matter because $G'$ will be accessed through the separator tree $T'$.

### Nested Dissection

We will need to compute with matrices. The arithmetic operations take place in ${\mathbb {Z}}_p$, where $p = \Theta (n^4)$ is prime. Thus, we work with numbers of $O(\log n)$-bits, and assuming a standard unit-cost model, we simply need to bound the number of arithmetic operations.

Let *A* be an $n\times n$ matrix. A Gaussian elimination step on row *i* is the following operation: for $j=i+1,\dots ,n$, add an appropriate multiple of row *i* to row *j* so that the element at position (*j*, *i*) becomes 0. Elimination on row *i* can be performed if the entry at position (*i*, *i*) is nonzero. Gaussian elimination on *A* consists of performing Gaussian elimination steps on rows $i=1,\dots , n-1$. This is equivalent to computing an *LU* decomposition of *A*, where *L* is a lower triangular matrix with units along the diagonal, and *U* is an upper triangular matrix. Gaussian elimination is performed *without pivoting* if, for all *i*, when we are about to do a Gaussian elimination step on row *i*, the entry at position (*i*, *i*) is non-zero. If Gaussian elimination is performed without pivoting, then the matrix is non-singular. (Pivoting is permuting the rows to ensure that the entry at position (*i*, *i*) is non-zero.)

Let $[n]=\{1,\dots , n\}$. The *representing graph* *G*(*A*) of an $n\hspace{1.111pt}{\times }\hspace{1.111pt}n$ matrix $A=(a_{i,j})_{i,j\in [n]}$ is

$$\begin{aligned}G(A)= \left( [n],\left\{ ij \in \left( {\begin{array}{c}[n]\\ 2\end{array}}\right) \;\Big |\; a_{i,j}\ne 0 \text { or }a_{j,i}\ne 0\right\} \right) .\end{aligned}$$

Let *T* be a separator tree for *G*(*A*). The row order of *A* is *consistent* with *T* if, whenever $t'$ is an ancestor of *t*, all the rows of $Z_t$ are before any row of $Z_{t'}$. We may assume that all the rows of $Z_t$ are consecutive. In particular, if the rows are ordered according to a post-order traversal of *T*, then the row order of *A* is consistent with *T*. A careful but simple revision of the nested dissection algorithm by Gilbert and Tarjan [14] leads to the following theorem.

#### Theorem 1.4

Let *A* be an $n\times n$ matrix such that the representing graph *G*(*A*) has bounded degree and assume that we are given a $(\gamma ,\beta ,\alpha )$-separator tree *T* for *G*(*A*), were $\gamma >0$, $0< \alpha <1$, and $1/2 \leqslant \beta < 1$ are constants. Furthermore, assume that the row order of *A* is consistent with *T* and that Gaussian elimination on *A* is done without pivoting. We can perform Gaussian elimination (without pivoting) on *A* and find a factorization $A=LU$ of *A* in $O\hspace{0.30548pt}(\gamma ^\omega n^{\beta \omega })$ time, where *L* is a lower triangular matrix with units along the diagonal and *U* is an upper triangular matrix.

For the proof of Theorem 3.2, we will need the following folklore lemma, whose proof we include for completeness.

#### Lemma 1.5

Let *A* be an $n\times n$ matrix, and $k \leqslant n$. Suppose that Gaussian elimination on the first *k* rows of *A* needs no pivoting. Then, we can perform Gaussian elimination on the first *k* rows of *A* with $O\hspace{0.33325pt}(n^2 k^{\omega -2})$ arithmetic operations.

#### Proof

Computing the inverse or performing Gaussian elimination for a $k\hspace{1.111pt}{\times }\hspace{1.111pt}k$ matrix takes $O(k^\omega )$ time (even if pivoting is needed), see, e.g., Bunch and Hopcroft [8], and Ibarra et al. [17].

Assume thatwhere $A_{1,1}$ is $k\times k$ and $A_{2,2}$ is $(n-k)\times (n-k)$. We want to perform Gaussian elimination without pivoting for the first *k* rows. First, we perform Gaussian elimination on the $k\times k$ matrix $A_{1,1}$. This takes $O(k^\omega )$ time, and we obtain two $k\times k$ matrices *L* and *U* such that $A_{1,1}=LU$, the matrix *L* is lower triangular with units along the diagonal, and the matrix *U* is upper triangular. Since we use no pivoting, *L* and *U* are non-singular. We also compute in $O(k^\omega )$ time the inverses $(A_{1,1})^{-1}$, $L^{-1}$, and $U^{-1}$. Then, we havewhich means that the second matrix on the right-side is the result of making Gaussian elimination for the first *k* rows of *A*. The products $A_{2,1}U^{-1}$, $L^{-1}A_{1,2}$, and $A_{2,1} (A_{1,1})^{-1} A_{1,2}$ can be computed in $O\hspace{0.31097pt}(n^2 k^{\omega -2})$ time by making at most $O(n^2/k^2)$ products of submatrices of size $k\times k$. $\square$

#### Proof of Theorem 3.2

We assume that the reader is familiar with some of the previous work on nested dissection to compute elimination orders for Gaussian elimination [14, 21]. Set $G = G(A)$. For each edge *ij* of *G*, if $i \in Z_t$ and $j \in Z_{t'}$, then either $t = t'$ or *t* and $t'$ have an ancestor–descendant relation in *T*.

For each node *t* of *T*, we eliminate all rows in $Z_t$ together, using block Gaussian eliminations. Since the row order is consistent with *T*, we have already eliminated all the rows of $V_t{\setminus } Z_t$, and we have not yet eliminated any row of $Z_{t'}$, for any ancestor $t'$ of *t*. For each node *t* of *T*, let $B_t$ be the set of vertices *j* that belong to some $Z_{t'}$, where $t'$ is an ancestor of *t* in *T*, such that there is an edge from *j* to some vertex of $V_t$. A vertex *j* of *G*(*A*) is affected by the elimination steps on the rows of $Z_t$ only if *j* belongs to $Z_t$ or to $B_t$. Thus, performing Gaussian elimination steps on the rows of $Z_t$ affects at most $|Z_t|+|B_t|$ rows and columns. Eliminating the rows of $Z_t$ affects the rows of $B_t$. However, when processing node *t*, we do not yet perform any elimination steps on the rows of $B_t$. Thus, we consider the submatrix with indices in $Z_t \cup B_t$, and we perform the elimination steps only on the rows of $Z_t$. By Lemma 3.3, this takes $O\hspace{0.33325pt}((|Z_t|+|B_t|)^2 |Z_t|^{\omega -2})$ time. It follows that the running time of the whole algorithm is

2 $$\begin{aligned} \sum _{t\in T}\hspace{0.83328pt}O\bigl ((|Z_t|+|B_t|)^2 |Z_t|^{\omega -2}\bigr )=\sum _{t\in T}\hspace{0.83328pt}O(|Z_t|^\omega ) + \sum _{t\in T}\hspace{0.83328pt}O\hspace{0.33325pt}(|B_t|^2|Z_t|^{\omega -2}). \end{aligned}$$

Since $|Z_t|\leqslant \gamma |V_t|^\beta$, the first sum is bounded as follows:

3 $$\begin{aligned} \sum _{t\in T}\hspace{0.83328pt}|Z_t|^\omega \leqslant \sum _{t\in T}\hspace{0.83328pt}\gamma ^\omega |V_t|^{\beta \omega }=\gamma ^\omega \sum _{t\in T}\hspace{0.83328pt}|V_t|^{\beta \omega }=O\hspace{0.33325pt}(\gamma ^\omega n^{\beta \omega } ), \end{aligned}$$

where in the last step we have used the assumption $\beta \omega >1$.

To bound the second sum, we first analyze $\Sigma = \sum _{t\in T} |B_t|^2$. For this, we follow Gilbert and Tarjan [14] almost verbatim. Let $L_\ell$ be the nodes of *T* at level $\ell$ and define $\Sigma _\ell = \sum _{t\in L_\ell } |B_t|^2$. (The root is at level 0.) Fix a level $\ell >0$. The sum $\Sigma _\ell$ is maximized if for each node $t'$ at level $\ell '<\ell$, all the edges with an endpoint in $t'$ and an endpoint at level at least $\ell$ are incident to the same subgraph $G[V_t]$ of $t\in L_\ell$. That is, to bound $\Sigma _\ell$, we can assume that all the edges incident to $Z_{t'}$ contribute to the same $B_t$, $t\in L_\ell$. For each $t\in L_ \ell$, let *s*(*t*) be the highest node of *T* with an edge going to $V_t$. Because of the assumption we made, the mapping $t \mapsto s(t)$ (from $L_\ell$ to $\bigcup _{\ell '\leqslant \ell } L_{\ell '}$) is injective. If $s(t)=t_0,t_1,\dots , t_a= t$ is the path in *T* from *s*(*t*) to *t*, then we have $|V_{t_i}|\leqslant \alpha ^i |V_{s(t)}|$ for each $i=0,\dots ,a$. Using that each vertex of each $Z_{t_i}$ has bounded degree, we get

$$\begin{aligned} |B_t|&\leqslant \sum _{i=0}^a O(1)|Z_{t_i}| \leqslant O(1)\sum _{i=0}^a \gamma \hspace{0.27771pt}|V_{t_i}|^\beta \leqslant O(\gamma )\sum _{i=0}^a\hspace{0.83328pt}(\alpha ^i |V_{s(t)}|)^\beta \\&\leqslant O(\gamma \hspace{0.28885pt}|V_{s(t)}|^\beta ) \sum _{i=0}^a\hspace{0.83328pt}(\alpha ^\beta )^i \leqslant O\biggl (\gamma \hspace{0.30655pt}|V_{s(t)}|^\beta \hspace{0.55542pt}\frac{1}{1-\alpha ^\beta }\biggr ). \end{aligned}$$

Since the map $t\mapsto s(t)$ is an injection (when $t\in L_\ell$), we have

$$\begin{aligned} \Sigma _\ell&=\sum _{t \in L_\ell } |B_t|^2\leqslant \sum _{t \in L_\ell } O\biggl (\gamma ^2|V_{s(t)}|^{2\beta }\hspace{0.55542pt}\frac{1}{(1-\alpha ^\beta )^2}\biggr )\\&\leqslant \!\sum _{s\in \bigcup _{\ell '\leqslant \ell }L_{\ell '}} \!\! O\biggl ( \gamma ^2|V_s|^{2\beta }\hspace{0.55542pt}\frac{1}{(1-\alpha ^\beta )^2}\biggr )=O\biggl ( \gamma ^2\hspace{0.55542pt}\frac{1}{(1-\alpha ^\beta )^2} \hspace{0.55542pt}\ell n^{2\beta }\biggr ), \end{aligned}$$

where in the last step we have used that the sets $V_s$, $s\in L_{\ell '}$, are pairwise disjoint subsets of [*n*] for each level $\ell '$, and $2\beta \geqslant 1$. For each $\ell$ and each $t\in L_\ell$, we have $|V_t|\leqslant \alpha ^\ell n$ and therefore $|Z_t|\leqslant \gamma (\alpha ^\ell n)^\beta$. This implies that

$$\begin{aligned} \sum _{t\in L_\ell } |B_t|^2 |Z_t|^{\omega -2}&\leqslant \sum _{t \in L_\ell } |B_t|^2 \bigl ( \gamma \hspace{0.33325pt}( \alpha ^\ell n )^\beta \bigr )^{\omega -2} =\gamma ^{\omega -2}(\alpha ^\ell n)^{\beta (\omega -2)}\sum _{t \in L_\ell } |B_t|^2 \\&\leqslant \gamma ^{\omega -2}(\alpha ^\ell n)^{\beta (\omega -2)} \cdot O\biggl ( \gamma ^2\hspace{0.55542pt}\frac{1}{(1-\alpha ^\beta )^2} \hspace{0.55542pt}\ell n^{2\beta }\biggr )\\&=O\biggl (\gamma ^{\omega }\frac{1}{(1-\alpha ^\beta )^2} \hspace{0.55542pt}n^{\beta \omega }\alpha ^\ell \ell \biggr ). \end{aligned}$$

Since $\sum _{\ell \geqslant 0} \alpha ^\ell \ell = \alpha /(1-\alpha )^2$, for $0<\alpha <1$, we get that

$$\begin{aligned} \sum _{t\in T}\hspace{0.83328pt}|B_t|^2 |Z_t|^{\omega -2}&=\sum _{\ell \geqslant 0} \sum _{t \in L_\ell } |B_t|^2 |Z_t|^{\omega -2}\leqslant \sum _{\ell \geqslant 0} O\biggl (\gamma ^{\omega }\frac{1}{(1-\alpha ^\beta )^2}\hspace{0.55542pt}n^{\beta \omega }\alpha ^\ell \ell \biggr )\\&=O\biggl (\frac{\alpha }{(1-\alpha ^\beta )^2(1-\alpha )^2}\hspace{0.55542pt}\gamma ^{\omega } n^{\beta \omega }\biggr ). \end{aligned}$$

Combining it with (3), we get from (2) that the total running time is $O\hspace{0.33325pt}((\alpha /(1-\alpha ^\beta )(1-\alpha )^2)\hspace{0.55542pt}\gamma ^{\omega }n^{\beta \omega })$. The theorem follows (in the statement of our theorem, we hide $\alpha$ in the *O*-notation, to avoid clutter and since the precise dependency is not important in our applications). $\square$

#### Remark 3.4

Mucha and Sankowski [28] noted that the result holds when *G*(*A*) is planar or, more generally, has recursive separators, using the approach by Lipton et al. [21] for nested dissection. This approach is based on the *strong* separator tree. Alon et al. [4, 35] showed that a similar result holds for graphs of bounded degree with recursive separators if one instead uses the nested dissection given by Gilbert and Tarjan [14]. In this case, we need bounded degree, but a weak separator tree suffices. Again, since we want to make the dependency on $\rho$ explicit and since the analysis in terms of matrix multiplication time does not seem to be written down in detail anywhere, we revise the method carefully.

#### Remark 3.5

Usually, the result is stated for symmetric positive definite matrices. Reindexing a symmetric positive definite matrix gives another symmetric positive definite matrix, and Gaussian elimination on such matrices can always be performed without pivoting. Thus, for positive semidefinite matrices, we do not need to assume that the row order is consistent with *T* because we can reorder the rows to make it consistent with *T*. However, Mucha and Sankowski [28] do need the general statement in their Sect. 4.2, and they mention this general case after their Theorem 13. Actually, they need it over ${\mathbb {Z}}_p$, where the concept of positive definiteness is not even defined!

### The Algorithm

Assume we have a graph *G* of $\mathbb {G}_\rho$ with *n* vertices and a geometric representation, i.e., geometric objects $\mathscr {U}$ of density at most $\rho$ such that *G* is a subgraph of $G_\mathscr {U}$. We want to compute a maximum matching for *G*. For this, we adapt the algorithm of Mucha and Sankowski [28]. We provide an overview of the approach, explain the necessary modifications, and emphasize the dependency on $\rho$ in the different parts of the algorithm.

Using Lemma 3.1, we get in $O(\rho n\log n)$ expected time a vertex-split graph $G'$ of *G* and a separator tree $T'$ for $G'$ such that

- the graph $G'$ has $\Theta (\rho n)$ vertices and edges;
- the maximum degree of $G'$ is at most 4;
- $T'$ is a $(\gamma =O(\rho ),\beta =1/2, \alpha )$-separator tree for $G'$, where $\alpha <1$ is a constant (independent of $\rho$ and *n*).

Since $G'$ is obtained from *G* by vertex splits, it suffices to find a maximum matching in $G'$. We set $m=|V(G')|=\Theta (\rho n)$, and we label the vertices of $G'$ from 1 to *m*. We consider the variables $X=(x_{ij})_{ij\in E(G')}$; i.e., each edge *ij* of *G* defines a variable $x_{ij}$. Consider the $m\times m$ symbolic matrix $A[X]=A[X](G')$, defined as follows:

$$\begin{aligned}(A[X])_{i,j}={\left\{ \begin{array}{ll} x_{ij}, &{} \hbox {if} \quad ij\in E(G')\quad \hbox {and}\quad i<j,\\ -x_{ij}, &{} \hbox {if} \quad ij\in E(G')\quad \hbox {and}\quad j<i,\\ 0,&{}\text {otherwise}. \end{array}\right. }\end{aligned}$$

The symbolic matrix *A*[*X*] is usually called the *Tutte matrix* of $G'$. It is known [30] that the rank of *A*[*X*] is twice the size of the maximum matching in $G'$. In particular, $G'$ has a perfect matching if and only if $\det \hspace{0.55542pt}(A[X])$ is not identically zero. Take a prime $p=\Theta (n^4)$, and substitute each variable in *A*[*X*] with a value from ${\mathbb {Z}}_p$, each chosen independently uniformly at random. Let *A* be the resulting matrix. Then, with high probability, ${{\,\mathrm{rank\hspace{0.44434pt}}\,}}(A)={{\,\mathrm{rank\hspace{0.44434pt}}\,}}(A[X])$, where on both sides we consider the rank over the field ${\mathbb {Z}}_p$ [30].

*From maximum matching to perfect matching*   Let $B=AA^T$. Then, *B* is symmetric, and the rank of *B* equals the rank of *A*. Note that $(B)_{i,j}$ is nonzero only if *i* and *j* share a neighbor in $G'$. Since $G'$ has bounded degree, from the separator tree $T'$ for $G'$, we can obtain a separator tree $T_B$ for the representing graph *G*(*B*). Since $T'$ was a $(\gamma =O(\rho ),\beta =1/2, \alpha )$-separator tree for $G'$, $T_B$ is a $(\gamma =O(\rho ),\beta =1/2, \alpha )$-separator tree for *G*(*B*), where the constant hidden in $O(\rho )$ is increased by the maximum degree in $G'$. Using Theorem 3.2, we obtain that Gaussian elimination can be done in *B* in $O\hspace{0.33325pt}(\gamma ^\omega m^{\omega /2})= O\hspace{0.33325pt}(\rho ^\omega (\rho n)^{\omega /2})=O\hspace{0.33325pt}(\rho ^{3\omega /2} n^{\omega /2})$ time, assuming that pivoting is not needed.

Mucha and Sankowski [28, Sect. 5] show how Gaussian elimination without pivoting can be used in *B* to find a collection of indices $W\subseteq [m]$ such that the centered matrix $(B)_{W,W}$, defined by rows and columns of *B* with indices in *W*, has the same rank as *B*. It follows that ${{\,\mathrm{rank\hspace{0.44434pt}}\,}}(A_{W,W})={{\,\mathrm{rank\hspace{0.44434pt}}\,}}(B_{W,W})$ and therefore $G'[W]$ contains a maximum matching of $G'$ that is a perfect matching in $G'[W]$ (with high probability). The key insight to find such *W* is that, if during Gaussian elimination in *B* we run into a 0 along the diagonal, then the whole row and column are 0, which means that they can be removed from the matrix without affecting the rank. We summarize.

#### Lemma 1.8

In time $O\hspace{0.33325pt}(\rho ^{3\omega /2} n^{\omega /2})$ we can find a subset *W* of vertices of $G'$ such that, with high probability, $G'[W]$ has a perfect matching that is a maximum matching in $G'$.

From now on, we can assume that $G'$ has a perfect matching. We keep denoting by $T'$ its separator tree, by *A* the matrix after substituting values of ${\mathbb {Z}}_p$ into *A*[*X*], and by *B* the matrix $AA^T$. (We can compute the tree $T'$ anew or we can reuse the same separator tree restricted to the subset of vertices.) Let $Z_r$ denote the set stored at the root *r* of $T'$. Thus, $Z_r$ is the first separator on $G'$. Let $N_r$ be the set $Z_r$ together with its neighbors in $G'$. Because $G'$ has bounded degree, we have $|N_r|=O(|Z_r|)=O(\rho m^{1/2})= O\hspace{0.33325pt}(\rho ^{3/2} n^{1/2})$.

Mucha and Sankowski show how to compute with *O*(1) Gaussian eliminations a matching $M'$ in $G'$ that covers all the vertices of $Z_r$ and is contained in some perfect matching of $G'$. There are two ingredients for this. The first ingredient is to use Gaussian elimination on the matrix $B = AA^T$ to obtain a decomposition $AA^T=LDL^T$, and then use (partial) Gaussian elimination on a matrix *C* composed of $L_{[m],N_r}$ and $A_{N_r,[m]\setminus N_r}$ to compute $(A^{-1})_{N_r,N_r}$. (Note that in general $(A^{-1})_{N_r,N_r}$ is different from $(A_{N_r,N_r})^{-1}$. Computing the latter is simpler, while computing the former is a major insight by Mucha and Sankowski [28, Sect. 4.2].) Interestingly, $T'$ is also a separator tree for the representing graph of this matrix *C*, and Gaussian elimination can be performed without pivoting. Thus, we can obtain in $O\hspace{0.33325pt}(\rho ^{\omega }m^{\omega /2})= O\hspace{0.33325pt}(\rho ^{3\omega /2} n^{\omega /2})$ time the matrix $(A^{-1})_{N_r,N_r}$. The second ingredient is that, once we have $(A^{-1})_{N_r,N_r}$, we can compute for any matching $M'$ contained in $G'[N_r]$ a maximal (with respect to inclusion) submatching $M'$ that is contained in a perfect matching of $G'$. This is based on an observation by Rabin and Vazirani [30] that shows how to find edges that belong to some perfect matching using the inverse matrix, and Gaussian elimination on the matrix $(A^{-1})_{N,N}$ to identify subsets of edges that together belong to some perfect matching. The matrix $(A^{-1})_{N_r,N_r}$ is not necessarily represented by a graph with nice separators, but it is of size $|N_r|\times |N_r|$. Thus, Gaussian elimination in $(A^{-1})_{N_r,N_r}$ takes $O(|N_r|^\omega )=O\hspace{0.33325pt}(\rho ^{3\omega /2} n^{\omega /2})$ time [28, Sect. 2.4].

Since the graph $G'$ has bounded maximum degree, making *O*(1) iterations of finding a maximal matching $M'$ in $G'[N_r]$, followed by finding a maximal subset $M''$ of $M'$ contained in a perfect matching of $G'$, and removing the vertices contained in $M'$ plus the edges of $M'\setminus M''$, gives a matching $M_*$ that covers $Z_r$ and is contained in a perfect matching of $G'$; see [28, Sect. 4.3]. The vertices of $M_*$ can be removed, and we recurse on both sides of $G' - V(M_*)\subset G' - Z_r$ using the corresponding subtrees of $T'$. The running time is $T(n)= O\hspace{0.33325pt}(\rho ^{3\omega /2} n^{\omega /2}) + T(n_1)+T(n_2)$, where $n_1,n_2\leqslant \alpha n$. This solves to $T(n) = O\hspace{0.33325pt}(\rho ^{3\omega /2} n^{\omega /2})$ because $\omega /2>1$. We summarize in the following result. If only the family $\mathscr {U}$ is given, first we use Lemma 2.1 to construct $G_\mathscr {U}$.

#### Theorem 1.9

Given a graph *G* of $\mathbb {G}_\rho$ with *n* vertices together with a family $\mathscr {U}$ of geometric objects with density $\rho$ such that *G* is a subgraph of $G_\mathscr {U}$, we can find in $O\hspace{0.33325pt}(\rho ^{3\omega /2} n^{\omega /2})$ time a matching in *G* that, with high probability, is maximum. In particular, for a family $\mathscr {U}$ of *n* geometric objects with density $\rho$, a maximum matching in $G_\mathscr {U}$ can be found in $O\hspace{0.33325pt}(\rho ^{3\omega /2} n^{\omega /2})$ time. The same holds for the bipartite or *k*-partite version of $G_\mathscr {U}$.

## Sparsification

Let $\mathscr {U}$ be a family of convex geometric objects in the plane such that each object of $\mathscr {U}$ contains a square of side length 1 and is contained in a square of side length $\Psi \geqslant 1$. Through the discussion we will treat $\Psi$ as a parameter. Our objective is to reduce the problem of computing a maximum matching in the intersection graph $G_\mathscr {U}$ to the problem of computing a maximum matching in $G_\mathscr {W}$ for some $\mathscr {W}\subseteq \mathscr {U}$ of small depth.

Let $P = {\mathbb {Z}}^2$ be the points in the plane with integer coordinates. Each square of unit side length contains at least one point of *P* and each square of side length $\Psi$ contains at most $(1+\Psi )^2=O(\Psi ^2)$ points of *P*. In particular, each object $U\in \mathscr {U}$ contains at least one and at most $O(\Psi ^2)$ points from *P*.

First we provide an overview of the idea. The objects intersected by a point $p \in P$ define a clique, and thus any even number of them defines a perfect matching. We show that, for each $p \in P$, it suffices to keep a few objects pierced by *p*, and we show how to obtain such a suitable subfamily. The actual number of objects to keep depends on $\Psi$, and whether the actual computation can be done efficiently depends on the geometric shape of the objects.

For each object $U \in \mathscr {U}$, we find the lexicographically smallest point in $P\cap U$. We assume that we have a primitive operation to compute $P \cap U$ for each object $U\in \mathscr {U}$ in $O\hspace{0.33325pt}(1+ |P\cap U|)= O(\Psi ^2)$ time. A simple manipulation of these incidences allows us to obtain the *clusters*

$$\begin{aligned} \mathscr {U}_p = \{ U\in \mathscr {U}\mid \hbox {} p~\hbox {lexicographically minimum in}~P\cap U\}, \end{aligned}$$

for all $p\in P$. Note that the clusters $\mathscr {U}_p$, for $p\in P$, form a partition of $\mathscr {U}$. This will be useful later. Clearly, the subgraph of $G_\mathscr {U}$ induced by $\mathscr {U}_p$ is a clique, for each $p\in P$. We will use the usual notation

$$\begin{aligned}E(\mathscr {U}_p,\mathscr {U}_q) =\{ UV\mid U\in \mathscr {U}_p, \, V\in \mathscr {U}_q,\,U\cap V\ne \emptyset \}\subseteq E(G_\mathscr {U}).\end{aligned}$$

The *pattern graph* $H=H(P,\Psi )$ has vertex set *P* and set of edges

$$\begin{aligned}E(H) = \{ pq \mid \Vert p-q\Vert _\infty \leqslant 2\Psi \} \subseteq \left( {\begin{array}{c}P\\ 2\end{array}}\right) .\end{aligned}$$

The use of the pattern graph is encoded in the following property: if $U\in \mathscr {U}_p$, $V\in \mathscr {U}_q$, and $U\cap V\ne \emptyset$, then $pq\in E(H)$. Indeed, if *U* and *V* intersect, then the union $U\cup V$ is contained in a square of side length $2\Psi$, and thus the $L_\infty$-distance between each $p\in P\cap U$ and $q\in P\cap V$ is at most $2\Psi$.

The definition of $H(P, \Psi )$ implies that the edge set of $G_\mathscr {U}$ is the disjoint union of $E(\mathscr {U}_p,\mathscr {U}_q)$, over all $pq\in E(H)$, and the edge sets of the cliques $G_{\mathscr {U}_p}$, over all $p\in P$. Thus, whenever $pq\notin E(H)$, there are no edges in $E(\mathscr {U}_p,\mathscr {U}_q)$.

Let $\lambda$ be the maximum degree of *H*. Note that $\lambda =O(\Psi ^2)$. The value of $\lambda$ is an upper bound on how many clusters $\mathscr {U}_q$ may interact with a single cluster $\mathscr {U}_p$. We will use $\lambda$ as a parameter to decide how many objects from each $\mathscr {U}_p$ are kept. We start with a simple observation.

### Lemma 1.10

There exists a maximum matching in $G_\mathscr {U}$ that, for all $pq\in E(H)$, contains at most one edge of $E(\mathscr {U}_p,\mathscr {U}_q)$.

### Proof

Let *M* be a maximum matching in $G_\mathscr {U}$ such that $\sum _{pq \in E(H)} |M \cap E(\mathscr {U}_p,\mathscr {U}_q)|$ is minimum. Suppose there is an edge $p_0q_0\in E(H)$ with $|M \cap E(\mathscr {U}_{p_0},\mathscr {U}_{q_0})|\geqslant 2$. Then we have two edges *UV* and $U'V'$ in $M\cap E(\mathscr {U}_{p_0},\mathscr {U}_{q_0})$, where $U,U'\in \mathscr {U}_{p_0}$ and $V,V'\in \mathscr {U}_{q_0}$. Since $UU'$ and $VV'$ are also edges in $G_\mathscr {U}$, we see that $M'= ( M\hspace{1.4444pt}{\setminus }\hspace{1.4444pt}\{ UV, U'V'\} ) \cup \{UU', VV'\}$ is a maximum matching in $G_\mathscr {U}$. We then have

$$\begin{aligned}|M'\cap E(\mathscr {U}_{p_0},\mathscr {U}_{q_0})| = |M\cap E(\mathscr {U}_{p_0},\mathscr {U}_{q_0})|-2,\end{aligned}$$

and

$$\begin{aligned}|M'\cap E(\mathscr {U}_p,\mathscr {U}_q)| = |M\cap E(\mathscr {U}_p,\mathscr {U}_q)|,\end{aligned}$$

for all $pq \in E(H)$, $pq \ne p_0q_0$. In this last statement, it is important that $\mathscr {U}_p$, $p\in P$, is a partition of $\mathscr {U}$, as otherwise $UU'$ could belong to some $E(\mathscr {U}_p,\mathscr {U}_q)$ or even $E(\mathscr {U}_{p_0},\mathscr {U}_{q_0})$. Hence, $\sum _{pq \in E(H)} |M' \cap E(\mathscr {U}_p,\mathscr {U}_q)|$ is strictly smaller than $\sum _{pq \in E(H)} |M \cap E(\mathscr {U}_p,\mathscr {U}_q)|$, a contradiction to our choice of *M*. The result follows. $\square$

Of course we do not know which object from the cluster $\mathscr {U}_p$ will interact with another cluster $\mathscr {U}_q$. We will explain how to get a large enough subset of cluster $\mathscr {U}_p$.

**Fig. 2:**
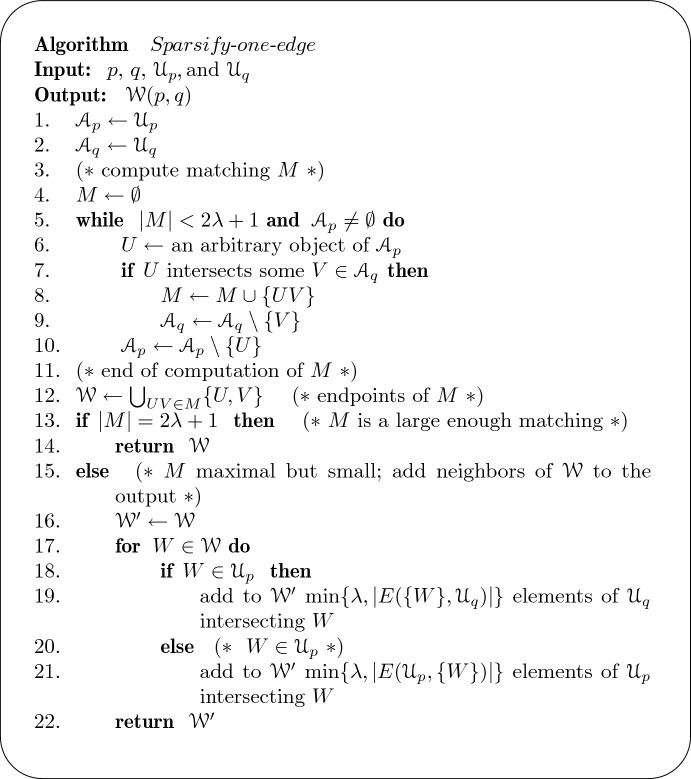
Algorithm *Sparsify-one-edge*

For each $pq\in E(H)$, we construct a set $\mathscr {W}(p,q)\subseteq \mathscr {U}_p\cup \mathscr {U}_q$ as follows. First, we construct a matching $M=M(p,q)$ in $E(\mathscr {U}_p,\mathscr {U}_q)$ such that *M* has $2\lambda +1$ edges or *M* has fewer than $2\lambda +1$ edges and is maximal in $E(\mathscr {U}_p,\mathscr {U}_q)$. For example, such a matching can be constructed incrementally. If *M* has $2\lambda +1$ edges, we take $\mathscr {W}(p,q)$ to be the endpoints of *M*. Otherwise, for each endpoint $U\in \mathscr {U}_p$ (resp. $V\in \mathscr {U}_q$) of *M*, we place *U* (resp. *V*) and $\lambda$ of its neighbors from $\mathscr {U}_q$ (resp. $\mathscr {U}_p$) into $\mathscr {W}(p,q)$. When *U* (resp. *V*) has fewer than $\lambda$ neighbors, we place all its neighbors in $\mathscr {W}(p,q)$. This finishes the description of $\mathscr {W}(p,q)$; refer to Algorithm $\textit{Sparsify-one-edge}$ in Fig. 2 for pseudo-code.

### Lemma 1.11

A maximum matching in

$$\begin{aligned}{\widetilde{G}} = \bigcup _{pq\in E(H)}\!\!\! G_{\mathscr {W}(p,q)}\cup \bigcup _{p\in P}G_{\mathscr {U}_p}.\end{aligned}$$

is a maximum matching in $G_\mathscr {U}$

### Proof

By Lemma 4.1, there is a maximum matching *M* in $G_\mathscr {U}$ such that for each $pq\in E(H)$, the matching *M* contains at most one edge from $E(\mathscr {U}_p,\mathscr {U}_q)$. Among all such maximum matchings, we choose one matching *M* that minimizes the number of edges $pq\in E(H)$ for which *M* contains an edge in $E(\mathscr {U}_{p},\mathscr {U}_q)$ that does not have both vertices in $\mathscr {W}(p,q)$. If there is no such edge $p_0q_0 \in E(H)$, then the lemma holds because such *M* is contained in ${\widetilde{G}}$. We show that this is the only possible case.

Suppose, for the sake of reaching a contradiction, that there exists $p_0q_0 \in E(H)$ such that *M* contains an edge *UV* with $U\in \mathscr {U}_{p_0}$, $V\in \mathscr {U}_{q_0}$, and $\{U, V\} \not \subset \mathscr {W}(p_0,q_0)$. Let $M'$ be the set of edges from *M* connecting different clusters, i.e., $M' = M\cap \bigcup _{pq\in E(H)} E(\mathscr {U}_p,\mathscr {U}_q)$. Let $M(p_0,q_0)$ be the matching in $E(\mathscr {U}_{p_0},\mathscr {U}_{q_0})$ used during the construction of $\mathscr {W}(p_0,q_0)$. We distinguish two cases:

$$|M(p_0,q_0)|$$
$= 2\lambda + 1$: Let $N_H(p)$ be the neighbors of *p* in *H*. Since *M* has at most one edge from $E(\mathscr {U}_{p},\mathscr {U}_q)$, for each $pq\in E(H)$, we obtain that $M'$ has at most $\lambda$ edges with an endpoint in $\mathscr {U}_{p_0}$ and at most $\lambda$ edges with an endpoint in $\mathscr {U}_{q_0}$, as $\lambda$ is the maximum degree of *H*. Thus, $M(p_0,q_0)$ contains at least one edge $U'V'$ whose endpoints are not touched by $M'$. We remove from *M* the edge *UV* and add the edge $U'V'$. If there was some edge $U'U''\in M\cap E(\mathscr {U}_{p_0},\mathscr {U}_{p_0})$, we also replace $U'U''$ by $UU''$ in *M*. If there was some edge $V'V''\in M\cap E(\mathscr {U}_{q_0},\mathscr {U}_{q_0})$, we also replace $V'V''$ by $VV''$ in *M*.
$$|M(p_0,q_0)|$$
$\leqslant 2\lambda$: In this case, $M(p_0,q_0)$ is a maximal matching in $E(\mathscr {U}_{p_0},\mathscr {U}_{q_0})$. In particular, one of *U* or *V* is covered by $M(p_0,q_0)$, as otherwise we could have added *UV* to $M(p_0,q_0)$. We consider the case when $U \in \mathscr {U}_{p_0}$ is covered by $M(p_0,q_0)$; the other case is symmetric. Then $U \in \mathscr {W}(p_0,q_0)$, $V\notin \mathscr {W}(p_0,q_0)$, and it follows that *U* has more than $\lambda$ neighbors in $\mathscr {U}_{q_0}$. Among the at least $\lambda$ neighbors of *U* in $\mathscr {W}(p_0,q_0)$, at most $\lambda - 1$ are covered by edges in $M'$. (Note that *V* is covered by $M'$, but *V* is not in $\mathscr {W}(p_0,q_0)$.) This means that there is some $V'\in \mathscr {U}_{q_0}\cap \mathscr {W}(p_0,q_0)$ such that $V'$ is not covered by $M'$ and $UV'\in E(\mathscr {U}_{p_0},\mathscr {U}_{q_0})$. We replace in *M* the edge *UV* by $UV'$. Moreover, if $V'V''$ is an edge of *M*, where necessarily $V''\in \mathscr {U}_{q_0}$, we replace in *M* the edge $V'V''$ by $VV''$.

In both cases, we can transform the maximum matching *M* into another maximum matching that contains one edge with both endpoints in $\mathscr {W}(p_0,q_0)$, no other edges of $E(\mathscr {U}_{p_0},\mathscr {U}_{q_0})\setminus E(G_{\mathscr {W}(p_0,q_0)})$, and the intersection of *M* with $E(\mathscr {U}_p,\mathscr {U}_q)$ has not changed, for all $pq\in E(H)\setminus \{ p_0 q_0\}$. This contradicts the choice of *M*, and the lemma follows. $\square$

### Lemma 1.12

The family of objects $\mathscr {W}=\bigcup _{pq\in E(H)}\mathscr {W}(p,q)$ has depth $O(\Psi ^8)$.

### Proof

Each $\mathscr {W}(p,q)$ has $O(\lambda ^2)$ elements, as the matching *M*(*p*, *q*) used for the construction of $\mathscr {W}(p, q)$ has $O(\lambda )$ edges, and each such edge may add $O(\lambda )$ more vertices to $\mathscr {W}(p,q)$. It follows that, for each $p\in P$, the family $\mathscr {W}$ contains at most

$$\begin{aligned}\sum _{q\in N_H(p)}\!\!|\mathscr {W}(p,q)| \leqslant \lambda \hspace{1.111pt}{ \cdot }\hspace{1.111pt}O(\lambda ^2) \leqslant O(\Psi ^6)\end{aligned}$$

objects from $\mathscr {U}_p$. In short, $|\mathscr {W}\cap \mathscr {U}_p|= O(\Psi ^6)$, for each $p\in P$. Fix a point $z \in {\mathbb {R}}^2$. Let *s* be a unit square that contains *z* and whose corners lie in *P*. For every object $U \in \mathscr {W}$ with $z \in U$, there is a square of side length $\Psi$ that contains *U* and at least one corner of *s*. Thus, each object *U* of $\mathscr {W}$ with $z \in U$ belongs to $\mathscr {U}_p$, for some $p\in P$ at $L_\infty$-distance at most $1+\Psi$ from *z*. It follows that *z* can only be contained in objects of $\mathscr {U}_p$ for $O(\Psi ^2)$ points $p\in P$, so the depth of *z* in $\mathscr {W}$ is at most

$$\begin{aligned}\sum _{\begin{array}{c} p\in P\\ \Vert z-p\Vert _\infty \leqslant 1+\Psi \end{array}}\!\!\!\!\! |\mathscr {W}\cap \mathscr {U}_p| \leqslant O(\Psi ^2)\cdot O(\Psi ^6)=O(\Psi ^8).\end{aligned}$$

Since *z* was arbitrary, the lemma follows. $\square$

### Theorem 1.13

Let $\mathscr {U}$ be a family of *n* geometric objects in the plane such that each object of $\mathscr {U}$ contains a square of side length 1 and is contained in a square of side length $\Psi$. Suppose that, for any $m \in {\mathbb {N}}$ and for any $p,q \in {\mathbb {Z}}^2$ with $|\mathscr {U}_p|+|\mathscr {U}_q|\leqslant m$, we can compute the sparsification $\mathscr {W}(p, q)$ as described above in time $T_spars (m)$, where $T_spars (m)=\Omega (m)$ is convex. In $O(\Psi ^2T_spars (n))$ time we can reduce the problem of finding a maximum matching in $G_\mathscr {U}$ to the problem of finding a maximum matching in $G_\mathscr {W}$ for some $\mathscr {W}\subseteq \mathscr {U}$ with maximum depth $O(\Psi ^8)$.

### Proof

For each $pq\in E(H)$, we find the sparsification $\mathscr {W}(p,q)$. Note that $\sum _{pq\in E(H)} (|\mathscr {U}_p|+|\mathscr {U}_q|)\leqslant \lambda n$, as each *p* contributes $\lambda$ summands. Hence, the computation of $\mathscr {W}(p,q)$, for all $pq\in E(H)$, takes time

$$\begin{aligned}\sum _{pq\in E(H)}\!\!\! O(T_spars (|\mathscr {U}_p|+|\mathscr {U}_q|)) =O(\lambda T_spars (n)) = O(\Psi ^2T_spars (n)).\end{aligned}$$

Consider the family $\mathscr {W}=\bigcup _{pq\in E(H)}\mathscr {W}(p,q)$. By Lemma 4.3, the family $\mathscr {W}$ has depth $O(\Psi ^8)$. By Lemma 4.2, it suffices to find a maximum matching in

$$\begin{aligned}{\widetilde{G}} =\bigcup _{pq\in E(H)}\!\!\! G_{\mathscr {W}(p,q)}\cup \bigcup _{p\in P}G_{\mathscr {U}_p},\end{aligned}$$

which is a subgraph of

4 $$\begin{aligned} G_{\mathscr {W}}\cup \bigcup _{p\in P}G_{\mathscr {U}_p}. \end{aligned}$$

Since each $G_{\mathscr {U}_p}$ is a clique and the vertices of $\mathscr {U}_p\setminus \mathscr {W}$ are not adjacent to any vertex outside $\mathscr {U}_p$ (in the graph (4)), we can just take maximum matchings within each $\mathscr {U}_p' = \mathscr {U}_p\setminus \mathscr {W}$. Here, we have to take care of the parity, as one vertex of $\mathscr {U}_p'$ may be left unmatched in $G_{\mathscr {U}_p'}$, but may be matched to some vertex of $\mathscr {U}_p\cap \mathscr {W}$. To handle this, for each $p \in P$ such that $|\mathscr {U}'_p|$ is odd, we move one element of $\mathscr {U}'_p$ to $\mathscr {W}$. Thus, we can assume that $|\mathscr {U}'_p|$ is even, for all $p \in P$. The additional elements in $\mathscr {W}$ may increase the depth of $\mathscr {W}$ by $O(\Psi ^2)$, which is negligible. Now, a maximum matching (4) is obtained by joining a maximum matching in $G_{\mathscr {W}}$ with maximum matchings in $G_{\mathscr {U}'_p}$, $p\in P$. The maximum matchings in $G_{\mathscr {U}'_p}$, $p \in P$, are trivial, because it is a clique on an even number of vertices. The result follows. $\square$

Our use of properties in the plane is very mild, and similar results hold in any space with constant dimension.

### Theorem 1.14

Let $d\geqslant 3$ be a constant. Let $\mathscr {U}$ be a family of *n* geometric objects in ${\mathbb {R}}^d$ such that each object of $\mathscr {U}$ contains a cube of side length 1 and is contained in a cube of side length $\Psi$. Suppose that, for any $m\in {\mathbb {N}}$ and for any $p,q \in {\mathbb {Z}}^d$ with $|\mathscr {U}_p|+|\mathscr {U}_q|\leqslant m$, we can compute the sparsification $\mathscr {W}(p, q)$ as described above in time $T_spars (m)$, where $T_spars (m)=\Omega (m)$ is convex. In $O(\Psi ^dT_spars (n))$ time we can reduce the problem of finding a maximum matching in $G_\mathscr {U}$ to the problem of finding a maximum matching in $G_\mathscr {W}$ for some $\mathscr {W}\subseteq \mathscr {U}$ with maximum depth $(1+\Psi )^{O(d)}$.

### Proof

The pattern graph *H* can be defined for ${\mathbb {Z}}^d$ also using the $L_\infty$-metric. Such pattern graph has maximum degree $O\hspace{0.33325pt}((1+\Psi )^d)=O(\Psi ^d)$. Lemmas 4.1 and 4.2 hold equally in this setting. Lemma 4.3 holds with an upper bound of $(1+\Psi )^{O(d)}$. The proof of Theorem 4.4 then applies. $\square$

As we mentioned in the introduction, for fat objects, bounded depth implies bounded density; see Har-Peled and Quanrud [15, Lemma 2.7]. If a convex object contains a cube of unit side length and is contained in a cube of side length $\Psi$, then it is $O(1/\Psi )$-fat; see van der Stappen et al. [32], where the parameter $1/\Psi$ goes under the name of thickness. Combining both results, one obtains that the relation between depth and density differs by a factor of $\Psi$. For fixed shapes, the depth and density differ by a constant factor.

## Efficient Sparsification

Now, we implement Algorithm $\textit{Sparsify-one-edge}$ (Fig. 2) efficiently. In particular, we must perform the test in line 7 and find the neighbors in line 19 (and the symmetric case in line 21). The shape of the geometric objects becomes relevant for this. First, we note that it suffices to obtain an efficient semi-dynamic data structure for intersection queries.

### Lemma 1.15

Suppose there is a data structure with the following properties: for any $m \in {\mathbb {N}}$ and for any $p, q \in {\mathbb {Z}}^2$ with $|\mathscr {U}_p|+|\mathscr {U}_q|\leqslant m$, we can maintain a set $\mathscr {A}_q\subseteq \mathscr {U}_q$ under deletions so that, for any query $U\in \mathscr {U}_p$, we either find some $V\in \mathscr {A}_q$ with $U \cap V \ne \emptyset$ or correctly report that no such *V* exists. Let $T_con (m)$ be the time to construct the data structure, $T_que (m)$ an upper bound on the amortized query time, and $T_del (m)$ be an upper bound on the amortized deletion time. Then, the running time of Algorithm $\textit{Sparsify-one-edge}$ (Fig. 2) for the input $(p,q,\mathscr {U}_p,\mathscr {U}_q)$ is $T_sparse (m)=O(T_con (m) +m T_que (m)+ \lambda ^2 T_del (m))$.

### Proof

First, we discuss the operations in lines 5–10 in Algorithm $\textit{Sparsify-one-edge}$ (Fig. 2). We maintain $\mathscr {A}_p$ as a linked list and $\mathscr {A}_q$ in the data structure from the lemma. This takes $O(T_con (m))$ time. Initially, $\mathscr {A}_p=\mathscr {U}_p$ and $\mathscr {A}_q=\mathscr {U}_q$. In each iteration of the while-loop, we query with *U* to either obtain some $V \in \mathscr {A}_q$ intersected by *U*, or correctly report that no object of $\mathscr {A}_q$ intersects *U*. If we get some *V* intersected by *U*, we remove *V* from $\mathscr {A}_q$ in $O(T_del (m))$ time. (Note that we have removed at most $2\lambda$ elements of $\mathscr {U}_q$ to obtain the current $\mathscr {A}_q$.) In either case, we remove *U* from $\mathscr {A}_p$, in *O*(1) time. The running time for this part is $O(mT_\text {que}(m)+ \lambda T_\text {del}(m))$.

Next, we discuss how to do line 19 in Algorithm $\textit{Sparsify-one-edge}$ (Fig. 2). We store $\mathscr {A}_q=\mathscr {U}_q$ in the data structure from the lemma. For each $W\in \mathscr {W}\cap \mathscr {U}_p$, we repeatedly query the data structure to find some $V\in \mathscr {A}_q$ that intersects *W*, and we remove this *V* from $\mathscr {A}_q$. We repeat this query-delete pattern in $\mathscr {A}_q$ with *W*, until we collect $\lambda$ neighbors of *W* or until we run out of neighbors. Thus, the query-deletion pattern happens at most $\lambda$ times, for each *W*. Having collected the data for *W*, we reverse all the deletions in $\mathscr {A}_q$, to obtain the original data structure for $\mathscr {A}_q=\mathscr {U}_q$, and we proceed to the next object of $\mathscr {W}\cap \mathscr {U}_p$. (We do not need insertions, as it suffices to undo the modifications that were made in the data structure.) In total, we repeat $O(|\mathscr {W}\cap \mathscr {U}_p|)=O(\lambda )$ times a pattern of $O(\lambda )$ queries and deletions followed by a reversal of all the operations. Thus, the running time is $T_con (m)$ to construct the data and $O(\lambda ^2 T_que (m)+ \lambda ^2 T_del (m))$ to handle the operations on the data structure. We can assume that $\lambda ^2\leqslant m$, as otherwise we do not need to run the sparsification and can take directly the whole set of objects.

Line 21 in Algorithm $\textit{Sparsify-one-edge}$ (Fig. 2) can be done in a similar way. The rest of the algorithms are elementary steps and bookkeeping. $\square$

### Disks in the Plane

When $\mathscr {U}$ consists of disks in the plane, we can use the data structure of Kaplan et al. [19], with a recent improvement by Liu [23], to sparsify an edge of the pattern graph. This leads to the following.

#### Proposition 1.16

Consider a family $\mathscr {U}$ of *n* disks in the plane with radii in $[1,\Psi ]$. In $O\hspace{0.33325pt}(\Psi ^6 n \log ^{4}\hspace{-0.7222pt}n)$ expected time, we can reduce the problem of finding a maximum matching in $G_\mathscr {U}$ to the problem of finding a maximum matching in $G_\mathscr {W}$ for some subfamily $\mathscr {W}\subseteq \mathscr {U}$ of disks with maximum depth $O(\Psi ^8)$.

#### Proof

Kaplan et al. [19] describe a data structure for additively weighted nearest-neighbor queries: maintain points $A = \{ a_1, \dots , a_n\} \subseteq {\mathbb {R}}^2$ in the plane, where each point $a_i$ has a weight $\omega _i\in {\mathbb {R}}$ associated to it. The data structure can handle insertions, deletions, and closest point queries (for a given $x\in {\mathbb {R}}^2$, return a point in $\arg \min _{a_i\in A} \omega _i+|x-a_i|$) in $O(\log ^{4}\hspace{-0.7222pt}n)$ amortized expected time.^7^

This data structure can be used to dynamically maintain a set $\mathscr {A}=\{D_1,\dots , D_n\}$ of disks so that, for a query disk *D*, we can either report one disk of $\mathscr {A}$ intersected by *D* or correctly report that no disk of $\mathscr {A}$ intersects *D*. Indeed, we store $\mathscr {A}$ as a set *A* of weighted points. Each disk $D_i$ is represented by its center $a_i$ with weight equal to its *negated* radius. If *x* is a point in the plane that lies outside the union of $\mathscr {A}$, the closest weighted point of *A* gives the first disk boundary that is touched by a growing disk centered at *x*. If *x* lies inside the union of $\mathscr {A}$, the closest weighted point gives the last boundary of a disk in $\mathscr {A}$ that contains *x* and that is touched by growing a disk around *x*. Thus, to answer a query for a disk *D*, we query for the weighted point $a_i\in A$ closest to the center of *D*, and then check whether *D* intersects $D_i$. Updates and queries take $O(\log ^{4}\hspace{-0.7222pt}n)$ amortized expected time.

Using Lemma 5.1, we conclude that $T_sparse (m)= O\hspace{0.33325pt}((m+\lambda ^2)\log ^{4}\hspace{-0.7222pt}{m})$ expected time. Recall that $\lambda ^2=O(\Psi ^4)$. Because of Theorem 4.4, we conclude that the reduc-tion takes time $O\hspace{0.33325pt}(\Psi ^2(n+\Psi ^4)\log ^{4}\hspace{-0.7222pt}n)=O\hspace{0.33325pt}(\Psi ^6n\log ^{4}\hspace{-0.7222pt}n)$ expected time. $\square$

Possibly, the method can be extended to homothets of a single object. For this one should consider the surfaces defined by weighted distances in the approach of Kaplan et al. [19]. Since the depth and the density of a family of disks are linearly related, Proposition 5.2 and Theorem 3.7 with $\rho =O(\Psi ^8)$ imply the following.

#### Theorem 1.17

Consider a family $\mathscr {U}$ of *n* disks in the plane with radii in the interval $[1,\Psi ]$. In $O\hspace{0.33325pt}(\Psi ^6 n \log ^{4}\hspace{-0.7222pt}n + \Psi ^{12\omega }n^{\omega /2})$ expected time, we can compute a matching in $G_\mathscr {U}$ that, with high probability, is maximum.

### Translates of a Fixed Convex Shape in the Plane

Now, suppose $\mathscr {U}$ consists of translates of a single convex object with non-empty interior in the plane. With an affine transformation, we ensure that the object is *fat*: the radii of the minimum enclosing disk and of the maximum enclosed disk are within a constant factor. Such a transformation is standard; e.g., [1, Lem. 3.2]. Thus, we may assume that $\Psi =O(1)$. We start with a standard lemma.

#### Lemma 1.18

Let $\mathscr {U}$ be a family of *n* translates of a convex object in the plane that are pierced by a given point *q*. The union of $\mathscr {U}$ can be computed in $O(n\log n)$ time.

#### Proof

The boundary of two translates of the same convex object intersect at most twice. This means that $\mathscr {U}$ is a *family of pseudodisks*. Let *q* be the given point that pierces all $U\in \mathscr {U}$. We assume that *q* belongs to the interior; otherwise it is necessary to make groups of objects and use *O*(1) points that intersect all the $U\in \mathscr {U}$.

Each $U \in \mathscr {U}$ defines a function $\delta _U:[0,2\pi ]\rightarrow {\mathbb {R}}$, where $\delta _U(\theta )$ is the length of the longest segment inside *U* with origin *q* and angle $\theta$ with some fixed axis. Since *q* is in the interior of *U*, the function $\delta _U(\,{\cdot }\,)$ is continuous. We can extend each function $\delta _U$ to the whole ${\mathbb {R}}$ by taking $\delta _U(\theta )=\delta (0)$, for $\theta \notin [0,2\pi ]$. The family $\{\delta _U\,|\,U\in \mathscr {U}\}$ of totally defined functions is a family of *pseudoparabolas*: the graphs of any two of them intersect at most twice.

The upper envelope of a family of *n* pseudoparabolas can be computed in $O\hspace{0.33325pt}(n \log n)$ time with a divide-and-conquer approach. First, we note that the upper envelope of *n* totally defined pseudoparabolas has at most $2n-1$ pieces. This is a standard property from the study of Davenport–Schinzel sequences. For the algorithm, we split the family $\mathscr {U}$ into two subfamilies $\mathscr {U}_1$ and $\mathscr {U}_2$ of roughly the same size, recursively compute the upper envelopes $g_1$ of $\mathscr {U}_1$ and $g_2$ of $\mathscr {U}_2$, and then compute the upper envelope of $g_1$ and $g_2$. If the upper envelopes are given as *x*-monotone curves, then the upper envelope of $g_1$ and $g_2$, which is the upper envelope of $\mathscr {U}$, is obtained in additional linear time. Since the merging step takes linear time, the whole algorithm takes $O\hspace{0.33325pt}(n \log n)$ time.

The maps $\delta _U$ do not need to be computed explicitly and the whole algorithm can actually be carried out with a rotational sweep around *q*. The transformation to consider the functions $\delta _U$ helps to bring it to familiar ground in computational geometry. $\square$

We will use the following lemma to “simulate” deletions. For this, we will keep a half-infinite interval of indices that contains the elements that are “deleted”.

#### Lemma 1.19

Let $\mathscr {U}=\{ U_1,\dots U_n\}$ be a family of *n* translates of a convex object in the plane that are pierced by a given point *q*. In $O(n\log ^2\hspace{-0.7222pt}n)$ time, we can construct a data structure for the following queries: given $x \in {\mathbb {R}}^2$ and a value $a \in \{1, \dots , n\}$, find the smallest $i\geqslant a$ such that $U_i$ contains *x*, or correctly report that *x* does not belong to $U_a\cup \dots \cup U_n$. The query time is $O(\log ^2\hspace{-0.7222pt}n)$.

#### Proof

We follow the standard approach for adding range capabilities to data structures [34]: we make a balanced binary search tree *T* whose leaves are $1,\dots , n$, from left to right. For each node $\nu$ of *T*, we define $C(\nu )$ as the set of indices stored at the leaves of the subtree rooted at $\nu$. The set $C(\nu )$ is a *canonical subset* of $\{ 1,\dots , n\}$.

For each node $\nu \in T$, we compute the region $R(\nu )=\bigcup _{i\in C(\nu )} U_i$. This can be done in $O(n \log n)$ time *for all* nodes $\nu$ of *T*. Indeed, the divide-and-conquer approach from the proof of Lemma 5.4 can be applied here. If a node $\nu$ has children $\nu _\ell$ and $\nu _r$, then $R(\nu )=\bigcup _{i\in C(\nu )} U_i$ can be computed in $O(|C(\nu )|)$ time from $R(\nu _\ell )=\bigcup _{i\in C(\nu _\ell )} U_i$ and $R(\nu _r)=\bigcup _{i\in C(\nu _r)} U_i$.

For each node $\nu$ of *T*, we preprocess the region $R(\nu )$ for point location queries. This takes $O(|C(\nu )|)$ time, because we just need the description of the boundary of $R(\nu )$ in a table. To decide whether a given point $x \in {\mathbb {R}}^2$ lies in $R(\nu )$, we make a binary search along the boundary of $R(\nu )$ for the arc of $R(\nu )$ that is intersected by the ray from *q* through *x*. This takes $O(\log n)$ time. This finalizes the preprocessing and the construction of the data structure.

Consider a query consisting of a point $x\in {\mathbb {R}}^2$ and an index *a*. We may assume that $a \in \{ 1,\dots ,n \}$. The set $\{a,a+1,\dots ,n\}$ can be expressed as the disjoint union of canonical subsets $C(\nu _1),\dots , C(\nu _k)$, where $k=O(\log n)$, indexed so that each element of $\nu _t$ is smaller than each element of $\nu _{t+1}$, for $t = 1, \dots , k - 1$. Making point location queries in $R(\nu _1),R(\nu _2),\dots$ we find the first index *j* such that $x\in R(\nu _j)$. This takes $O(\log ^2\hspace{-0.7222pt}n)$, as we make $O(\log n)$ point location queries. Then, we search the subtree of *T* rooted at $\nu _j$ for the leftmost leaf *i* with $x\in \mathscr {U}_i$. This is easy: if we are at some internal node $\nu$ with left child $\nu _\ell$ and right child $\nu _r$, we query the point location data structure at $\nu _\ell$ to determine whether $x\in R(\nu _\ell )$. If $x\in R(\nu _\ell )$, we continue to $\nu _\ell$. Otherwise, *x* must be in $R(\nu _r)$, as $x\in R(\nu )$, and we go to $\nu _r$. This search makes $O(\log n)$ queries to the point location structures, and thus takes $O(\log ^2\hspace{-0.7222pt}n)$ time. $\square$

#### Lemma 1.20

Let $\mathscr {U}_q=\{ V_1,\dots , V_n\}$ be a family of *n* translates of a convex object in the plane that are pierced by a given point *q*. Let $U_0$ be a convex object. In $O(n\log ^2\hspace{-0.7222pt}n)$ time, we can construct a data structure for the following type of queries: given a translate *U* of $U_0$ and a value *a*, find the smallest $i\geqslant a$ such that *U* intersects $V_i$, or correctly report that *U* does not intersect $V_a\cup \dots \cup V_n$. Each query can be answered in $O(\log ^2\hspace{-0.7222pt}n)$ time.

#### Proof

Applying a translation, we may assume that $U_0$ contains the origin. For each $V_i\in \mathscr {U}_q$, let $W_i=V_i\oplus U_0 = \{ v-u\mid v\in V_i, \, u\in U_0\}$ be the Minkowski sum of $V_i$ and $-U_0$. For each translation $\tau$, we have that $\tau (U_0)$ intersects $V_i$ if and only if $\tau \in W_i$. All the sets $W_1,\dots ,W_n$ contain *q* because the origin belongs to $U_0$. Thus, we can construct the data structure of Lemma 5.5 for $\{W_1,\dots ,W_n\}$. For a query *U* and *a*, we find the translation $\tau$ such that $U=\tau (U_0)$, and then find the smallest $i\geqslant a$ such that $\tau \in W_i$, which also tells the smallest $i\geqslant a$ such that *U* intersects $V_i$. $\square$

Lemma 5.6 can be used to make queries and simulate deletions.

#### Proposition 1.21

Consider a family $\mathscr {U}$ of *n* translates of a convex object with non-empty interior in the plane. In $O(n\log ^2\hspace{-0.7222pt}n)$ time, we can reduce the problem of finding a maximum matching in $G_\mathscr {U}$ to the problem of finding a maximum matching in $G_\mathscr {W}$ for some subfamily $\mathscr {W}\subseteq \mathscr {U}$ with maximum depth *O*(1).

#### Proof

As mentioned above, we may make an affine transformation, so that, after the transformation, we have $\Psi =O(1)$ [1, Lemma 3.2]. Consider an edge *pq* of the pattern graph. We use the algorithm described in Lemma 5.1, but with a slight modification. We order the objects of $\mathscr {U}_q$ as $\{ V_1,\dots ,V_m\}$ and use the data structure of Lemma 5.6 to store them. At the start we set $a=1$. Whenever we want to query $\mathscr {A}_q$ with *U*, we query the data structure with *U* and the current *a*. If the data structure returns $V_i$, we set $a=i+1$ for future queries to the data structure. In this way, each time we query the data structure, we find a new element of $\mathscr {U}_q$ that has not been reported before. Thus, we obtain the same running time as in Lemma 5.1 with $T_con (m)=O\hspace{0.27771pt}(m\log m)$, $T_que (m)=O\hspace{0.29999pt}(\log ^2\hspace{-0.7222pt}m)$ and $T_del (m)=O(1)$. Therefore $T_sparse (m)=O\hspace{0.33325pt}(m \log ^2\hspace{-0.7222pt}m)$, and the result follows from Theorem 4.4. $\square$

Combining Proposition 5.7 and Theorem 3.7 we obtain the following.

#### Theorem 1.22

Consider a family $\mathscr {U}$ of translates of a convex object with non-empty interior in the plane. In $O(n^{\omega /2})$ time we can find a matching in $G_\mathscr {U}$ that, with high probability, is maximum.

If $\mathscr {U}$ consists of unit disks, the sparsification can be done slightly faster using a semi-dynamic data structure by Efrat et al. [11], which has $T_con (m)=O\hspace{0.30548pt}(m\log m)$, $T_del (m)=O(\log m)$, and $T_que (m) = O(\log m)$. However, the current bottleneck is the computation of the maximum matching *after* the sparsification. Thus, improving the sparsification in the particular case of unit disks does not lead to an improved final algorithm.

Proposition 5.7 and Theorem 5.8 also holds if we have translations of *O*(1) different convex objects (with nonempty interiors). Indeed, the data structure of Lemma 5.6 can be made for each pair of different convex shapes. In this case, the constant $\Psi$ depends on the shapes, namely the size of the largest square that we can place inside each of the convex shapes and the size of the smallest square that can be used to cover each of the convex shapes. Also, the relation between the depth and the density depends on the shapes. However, for a fixed set of *O*(1) shapes, both values are constants that depend on the shapes.

#### Theorem 1.23

Suppose we are given a set ${\mathscr {A}}$ of *O*(1) different convex objects in the plane with non-empty interiors. Let $\mathscr {U}$ be a family that contains *n* translates of objects from ${\mathscr {A}}$. Then, we can find in $O(n^{\omega /2})$ time a matching in $G_\mathscr {U}$ that is maximum with high probability. Here, the constant in the *O*-notation depends on ${\mathscr {A}}$.

### Axis-Parallel Objects

A *box* is the Cartesian product of intervals. Combining standard data structures for orthogonal range searching [6, Sects. 5.4 and 10.3] one obtains the following results.

#### Proposition 1.24

Let $d\geqslant 2$ be an integral constant. Consider a family $\mathscr {U}$ of *n* boxes in ${\mathbb {R}}^d$ such that each box of $\mathscr {U}$ contains a cube of side length 1 and is contained in a cube of side length $\Psi$. In $O(\Psi ^dn \log ^{O(d)}\hspace{-0.7222pt}n)$ time we can reduce the problem of finding a maximum matching in $G_\mathscr {U}$ to the problem of finding a maximum matching in $G_\mathscr {W}$, for some $\mathscr {W}\subseteq \mathscr {U}$ with maximum depth $(1+\Psi )^{O(d)}$.

#### Proof

Edelsbrunner and Maurer [10] show a general approach to provide a data structure to dynamically maintain a set of boxes and handle the following queries: given a box *b*, report all the boxes in the data structure that intersect *b*. The construction time is $O\hspace{0.30548pt}(n\log ^d\hspace{-0.7222pt}n)$, each update (deletion/insertion) takes $O(\log ^d\hspace{-0.7222pt}n)$ time, and each query takes $O\hspace{0.27771pt}(k+\log ^d\hspace{-0.7222pt}n)$, where *k* is the size of the output. The data structure is a combination of segment and range trees. Such a data structure can easily be modified to report a single element intersecting the query box *b* in $O(\log ^d\hspace{-0.7222pt}n)$ time. In fact, better results can be obtained with more advanced techniques, but we feel that discussing them is not relevant here. (Also, we only need deletions, which makes it simpler, as in the relevant trees we can just mark some vertices as deleted.) Using Lemma 5.1 and Theorem 4.5, we obtain the result. $\square$

For $d=2$, we can combine Theorem 3.7 and Proposition 5.10. Since we have assumed $\omega >2$, the $O\hspace{0.33325pt}(n\log ^{O(d)}\hspace{-0.7222pt}n)$ term is asymptotically smaller than $O(n^{\omega /2})$, and we obtain the following.

#### Theorem 1.25

Given a family $\mathscr {U}$ of *n* boxes in ${\mathbb {R}}^2$ such that each object of $\mathscr {U}$ contains a square of side length 1 and is contained in a square of side length $\Psi$, we can compute in $(1+\Psi )^{O(1)} n^{\omega /2}$ time a matching in $G_\mathscr {U}$ that, with high probability, is a maximum matching.

Consider now the case $d\geqslant 3$. The set $\mathscr {W}$ that we obtain from Proposition 5.10 has depth and density $\rho =(1+\Psi )^{O(d)}$, and therefore the graph $G_\mathscr {W}$ has $O(\rho n)$ edges; see Lemma 2.1. We can thus use the algorithm of Micali and Vazirani [25, 33], which takes $O(\sqrt{n}\hspace{0.66666pt}|E(G_\mathscr {W})|) = (1+\Psi )^{O(d)} n^{3/2}$ time. We summarize.^8^

#### Corollary 1.26

Let $d\geqslant 3$ be an integral constant. Given a family $\mathscr {U}$ of *n* boxes in ${\mathbb {R}}^d$ such that each object of $\mathscr {U}$ contains a cube of side length 1 and is contained in a cube of side length $\Psi$, we can compute in $(1+\Psi )^{O(d)} n^{3/2}$ time a maximum matching in $G_\mathscr {U}$.

### Congruent Balls in $d\geqslant 3$ Dimensions

Consider now the case of congruent balls in ${\mathbb {R}}^d$, for constant $d\geqslant 3$. Note that $\lambda =O(1)$ in this case. We use the dynamic data structure by Agarwal and Matoušek [2] for the sparsification. For each *m* with $n\leqslant m\leqslant n^{\lceil d/2\rceil }$, the data structure maintains *n* points in ${\mathbb {R}}^d$, answers *O*(*n*) queries for closest point, and supports $O(\lambda ^2)$ updates in

$$\begin{aligned} O\biggl (m^{1+\varepsilon }+ \lambda ^2 \hspace{0.55542pt}\frac{m^{1+\varepsilon }}{n} +n\hspace{0.55542pt}\frac{n \log ^3\hspace{-0.7222pt}n}{ m^{1/\lceil d/2\rceil }}\biggr ) \end{aligned}$$

time. Here $\varepsilon > 0$, is an arbitrary constant whose choice affects to the constants hidden in the *O*-notation. For $d \in \{3, 4\}$, this running time is

$$\begin{aligned} O\biggl (m^{1+\varepsilon }+ \lambda ^2 \hspace{0.55542pt}\frac{m^{1+\varepsilon }}{n} +n\hspace{0.55542pt}\frac{n \log ^3\hspace{-0.7222pt}n}{m^{1/2}}\biggr ). \end{aligned}$$

Setting $m=n^{4/3}$, we get a running time of $O\hspace{0.33325pt}(n^{4/3+\varepsilon } + \lambda ^2 n^{1/3+\varepsilon })=O(n^{4/3+\varepsilon })$ to handle *O*(*n*) queries and $O(\lambda ^2)=O(1)$ updates. Using this in Lemma 5.1 and Theorem 4.5, we get the following result.

#### Proposition 1.27

Consider a family $\mathscr {U}$ of *n* unit balls objects in ${\mathbb {R}}^d$, for $d \in \{3, 4\}$. In $O(n^{4/3+\varepsilon })$ time, we can reduce the problem of finding a maximum matching in $G_\mathscr {U}$ to the problem of finding a maximum matching in $G_\mathscr {W}$ for some $\mathscr {W}\subseteq \mathscr {U}$ with maximum depth *O*(1).

For the resulting set $\mathscr {W}$ with depth *O*(1), it is better to use the algorithm of Micali and Vazirani [25, 33]. Note that $G_\mathscr {W}$ is sparse, and thus has *O*(*n*) edges. Therefore, a maximum matching in $G_\mathscr {W}$ can be computed in $O(n^{3/2})$ time. In summary, we spend $O(n^{4/3+\varepsilon })$ for the sparsification and $O(n^{3/2})$ for computing the matching in the sparsified setting.^9^

For $d > 4$, we set $m=n^{{2 \lceil d/2\rceil }/({1+ \lceil d/2\rceil })}$. The running time for the sparsification is then

5 $$\begin{aligned} O\bigl (n^{{2 \lceil d/2\rceil }/{(1+ \lceil d/2\rceil )}+\varepsilon }\bigr ). \end{aligned}$$

For each constant *d*, the resulting instance $G_\mathscr {W}$ has *O*(*n*) edges. For $d=5,6$, the running time of the sparsification is $O(n^{3/2+\varepsilon })$. However, after the sparsification, we have a graph with *O*(*n*) edges, and we can use the algorithm of Micali and Vazirani [25, 33], which takes $O(n^{3/2})$ time. Thus, for $d\geqslant 5$, the running time is dominated by the sparsification.

#### Theorem 1.28

Let $d\geqslant 3$ be a constant. Consider a family $\mathscr {U}$ of congruent balls in ${\mathbb {R}}^d$. For $d=3,4$, we can find in $O(n^{3/2})$ time a maximum matching in $G_\mathscr {U}$. For $d\geqslant 5$, we can find in time (5) a maximum matching in $G_\mathscr {U}$, for each $\varepsilon >0$.

## Conclusion

We have proposed the density of a geometric intersection graph as a parameter for the maximum matching problem, and we showed that it can be fruitful in obtaining efficient matching algorithms. Then, we presented a sparsification method that lets us reduce the general problem to the case of bounded density for several interesting classes of geometric intersection graphs. In our sparsification method, we did not attempt to optimize the dependency on the radius ratio $\Psi$. It may well be that this can be improved by using more advanced grid-based techniques. Furthermore, our sparsification needs the complete intersection graph and does not apply to the bipartite setting. Here, we do not know of a method to reduce the general case to bounded density. In general, the complexity of the matching problem is wide open. To the best of our knowledge, there are no (even weak) superlinear lower bounds for the (static) matching problem in general graphs.

## Footnotes

1 In the literature, it is more common to assume $\omega \geqslant 2$. We adopt the stronger assumption $\omega > 2$ because it simplifies the bounds. If $\omega =2$ is allowed, then the running times that we state have additional logarithmic factors.

2 Note that by definition, any vertex-induced subgraph of a geometric intersection graph of density at most $\rho$ is also a geometric intersection graph of density at most $\rho$. Thus, any graph in $\mathbb {G}_\rho$ is obtained by omitting edges from some geometric intersection graph of density at most $\rho$ with the same vertex set.

3 In a previous version of this paper, we claimed that Mądry’s algorithm also applies to general (non-bipartite) graphs. However, this does not seem to be correct. As a consequence, we have updated the statements of Corollary 5.12 and Theorem 5.14.

4 A graph family is called *hereditary* if it is closed under taking subgraphs.

5 Note that here we do not consider subgraphs of $G_\mathscr {U}$; we need the whole graph $G_\mathscr {U}$.

6 The original algorithm is described for line segments, but assuming appropriate geometric primitives, it also applies to continuous, *x*-monotone curves in the plane.

7 The running time in [19] has more logarithmic factors, but recently Liu [23] presented an improved construction of shallow cuttings that leads to the claimed result.

8 In a previous version, we invoked Mądry’s algorithm and claimed a better running time with exponent 10/7. However, this algorithm does not seem to apply here, so we fall back on the Micali–Vazirani algorithm to obtain an exponent of 3/2.

9 Also here, we previously claimed a better exponent of 10/7, which was based on an incorrect invocation of Mądry’s algorithm.
